# Supplementary material for: Systematic Investigation of Phosphate Decomposition and Soil Fertility Modulation by the Filamentous Fungus Talaromyces nanjingensis
Source: Microorganisms. 2025 Jul 3;13(7):1574. doi: 10.3390/microorganisms13071574 (PMC12301037; doi:10.3390/microorganisms13071574)
Supplement: Supplementary file 1 [file microorganisms-13-01574-s001.zip › Table S1.pdf]

Tab. S1 Genes associated with phosphate-solubilizing or phosphate-related found in *T. nanjingensis* (detailed information version)

Genes and Seq ID		Gene function annotation
**Organic acid**		
1	*Tspbctg00000001G00023830.1*	*GO:0006082//organic acid metabolic process`biological_process;*
2	*Tspbctg00000000G00000690.1*	*GO:0006082//organic acid metabolic process`biological_process;*
3	*Tspbctg00000000G00014510.1*	*GO:0006082//organic acid metabolic process`biological_process;*
4	*Tspbctg00000003G00058590.1*	*GO:0006082//organic acid metabolic process`biological_process;*
5	*Tspbctg00000000G00010340.1*	*InP/2,5-diketo-d-gluconic acid reductase;*
6	*Tspbctg00000004G00071520.1*	*InP/bicupin_oxalic: bicupin, oxalate decarboxylase family;*<br>*TIGR03404.1//bicupin_oxalic`JCVI: bicupin, oxalate decarboxylase family (Provisional)*
7	*Tspbctg00000000G00005530.1*	*K16066//ydfG`3-hydroxy acid dehydrogenase/malonic semialdehyde reductase [EC:1.1.1.381 1.1.1.-];*
8	*Tspbctg00000005G00082040.1*	*Nr/PCH00005.1//C4-dicarboxylate transporter/malic acid transport protein;*<br>*tr|A0A2H3IE11|A0A2H3IE11_9EURO//C4-dicarboxylate transporter/malic acid transport protein {ECO:0000313|EMBL:PCH00005.1} OS=Penicillium sp. 'occitanis' ORFNames=PENO1_050100 {ECO:0000313|EMBL:PCH00005.1} PE=3;*
9	*Tspbctg00000005G00082050.1*	*tr|A0A2H3IE11|A0A2H3IE11_9EURO//C4-dicarboxylate transporter/malic acid transport protein {ECO:0000313|EMBL:PCH00005.1} OS=Penicillium sp. 'occitanis' ORFNames=PENO1_050100 {ECO:0000313|EMBL:PCH00005.1} PE=3;*
**Acid phosphatase**		
1	*Tspbctg00000002G00044030.1*	*K01078//PHO`acid phosphatase [EC:3.1.3.2];*<br>*Nr/KAF3399491.1//Acid phosphatase;*
2	*Tspbctg00000002G00042420.1*	*InP/HP_HAP_like;Histidine phosphatase superfamily (branch 2);3-PHYTASE B;Phosphoglycerate mutase-like;Acid_Ptase;Histidine acid phosphatases active site signature.;Histidine acid phosphatases phosphohistidine signature.;Multiple inositol polyphosphate phosphatase-related;*<br>*Ref/XP_002150501.1//acid phosphatase, putative;*
3	*Tspbctg00000002G00042840.1*	*InP/Phosphatidic acid phosphatase type 2/haloperoxidase; PAP2_containing_1_like;Acid phosphatase/Vanadium-dependent haloperoxidase; PAP2 superfamily;acid_phosph_2;Lipid phosphate phosphatase;PAP2 Domain protein (AFU_ORTHOLOGUE AFUA_4G08970);*
4	*Tspbctg00000002G00041030.1*	*K01078//PHO`acid phosphatase [EC:3.1.3.2];*<br>*InP/Acid phosphatase phoa (AFU_ORTHOLOGUE AFUA_1G03570);Alkaline Phosphatase, subunit A;Phosphoesterase*

		family;Non-specific phospholipase C4-related;
5	*Tspbctg00000000G00020390.1*	*InP/Phosphatidic acid phosphatase type 2/haloperoxidase;Palmitoyl-protein thioesterase/dolichyldiphosphatase 1;Acid phosphatase/Vanadium-dependent haloperoxidase;PAP2 superfamily;PAP2_dolichyldiphosphatase;Dolichyldiphosphatase 1;acid_phosph_2;*
6	*Tspbctg00000001G00024240.1*	*K01078//PHO`acid phosphatase [EC:3.1.3.2];<br>tr|A0A2H3I9R4|A0A2H3I9R4_9EURO//Purple acid phosphatase {ECO:0000256|RuleBase:RU361203} EC=3.1.3.2 {ECO:0000256|RuleBase:RU361203} OS=Penicillium sp. 'occitanis' ORFNames=PENO1_062130 {ECO:0000313|EMBL:PCG97663.1} PE=3;<br>GO:0003993//acid phosphatase activity`molecular_function;<br>PF16656.8//Pur_ac_phosph_N`Purple acid Phosphatase, N-terminal domain;PF00149.31//Metallophos`Calcineurin-like phosphoesterase;PF14008.9//Metallophos_C`Iron/zinc purple acid phosphatase-like protein C;<br>InP/Iron/zinc purple acid phosphatase-like protein C;Purple acid phosphatase, N-terminal domain;Acid_Ptase_Asper;Calcineurin-like phosphoesterase;Acid phosphatase related;Metallo-dependent phosphatases;Purple acid phosphatase;MPP_PAPs;*
7	*Tspbctg00000005G00074090.1*	*K01078//PHO`acid phosphatase [EC:3.1.3.2];<br>Nr/KAF3387880.1//Acid phosphatase;*
8	*Tspbctg00000003G00049580.1*	*tr|A0A2H3IZ54|A0A2H3IZ54_9EURO//Phosphatidic acid phosphatase type 2/haloperoxidase {ECO:0000313|EMBL:PCH05311.1} OS=Penicillium sp. 'occitanis' ORFNames=PENO1_023060 {ECO:0000313|EMBL:PCH05311.1} PE=3;<br>InP/PAP2 superfamily;Phosphatidic acid phosphatase type 2/haloperoxidase;acid_phosph_2;RE23632P;Lipid phosphate phosphatase; PAP2_containing_1_like;Acid phosphatase/Vanadium-dependent haloperoxidase;*
9	*Tspbctg00000002G00047930.1*	*K01078//PHO`acid phosphatase [EC:3.1.3.2];<br>InP/Acid_Ptase;HP_HAP_like;Multiple inositol polyphosphate phosphatase-related;Acid phosphatase, putative (AFU_ORTHOLOGUE AFUA_6G11330)-related;Histidine phosphatase superfamily (branch 2);Histidine acid phosphatases phosphohistidine signature.;Phosphoglycerate mutase-like;*
10	*Tspbctg00000000G00002060.1*	*tr|A0A2H3I933|A0A2H3I933_9EURO//Phosphatidic acid phosphatase type 2/haloperoxidase {ECO:0000313|EMBL:PCG97303.1} OS=Penicillium sp. 'occitanis' ORFNames=PENO1_063880 {ECO:0000313|EMBL:PCG97303.1} PE=4;<br>InP/PAP2 superfamily;Acid phosphatase/Vanadium-dependent haloperoxidase;Sphingosine-1-phosphate phosphohydrolase;Dihydrosphingosine 1-phosphate phosphatase lcb3-related;acid_phosph_2;PAP2_SPPase1;Phosphatidic acid phosphatase type 2/haloperoxidase;*
11	*Tspbctg00000007G00092920.1*	*InP/Phosphoglycerate mutase-like;HP_HAP_like;3-Phytase B;Histidine acid phosphatases phosphohistidine*

		signature.;Multiple inositol polyphosphate phosphatase-related;Acid_Ptase;Histidine phosphatase superfamily (branch 2);
12	*Tspbctg00000007G00093540.1*	*K01078//PHO`acid phosphatase [EC:3.1.3.2];*<br>*InP/HP_HAP_like;Multiple inositol polyphosphate phosphatase-related;HP;Acid phosphatase pho11-related;Phosphoglycerate mutase-like;Histidine phosphatase superfamily (branch 2);*<br>*Ref/XP_002148872.1//repressible acid phosphatase precursor, putative;*
13	*Tspbctg00000003G00057840.1*	*TIGR03396.1//PC_PLC`JCVI: phospholipase C, phosphocholine-specific;TIGR03397.1//acid_phos_Burk`JCVI: acid phosphatase;*
14	*Tspbctg00000001G00027590.1*	*K01078//PHO`acid phosphatase [EC:3.1.3.2];*<br>*KOG3720`Lysosomal & prostatic acid phosphatases`Lipid transport and metabolism;*<br>*InP/Phosphoglycerate mutase-like;Histidine phosphatase superfamily (branch 2);Acid phosphatase-related;HP_HAP_like;Lysophosphatidic acid phosphatase type 6;*
15	*Tspbctg00000007G00099440.1*	*TIGR03396.1//PC_PLC`JCVI: phospholipase C, phosphocholine-specific;TIGR03397.1//acid_phos_Burk`JCVI: acid phosphatase;*
16	*Tspbctg00000005G00083920.1*	*K01078//PHO`acid phosphatase [EC:3.1.3.2];*<br>*InP/Acid phosphatase-related;Phosphoglycerate mutase-like;*
17	*Tspbctg00000006G00085240.1*	*K14379//ACP5`tartrate-resistant acid phosphatase type 5 [EC:3.1.3.2];*<br>*Nr/KAF3397623.1//Tartrate-resistant acid phosphatase type 5;*<br>*InP/Calcineurin-like phosphoesterase;Tartrate-resistant acid phosphatase type 5;Metallo-dependent phosphatases;*<br>*Ref/XP_002152238.1//tartrate-resistant acid phosphatase type 5 precursor, putative;*
18	*Tspbctg00000002G00040730.1*	*InP/Metallo-dependent phosphatases;Calcineurin-like phosphoesterase;Inactive purple acid phosphatase 16-related;MPP_Dcr2;*
19	*Tspbctg00000001G00034350.1*	*InP/Acid phosphatase-related;Acid phosphatase, putative (AFU_ORTHOLOGUE AFUA_3G14570)-related;Phosphoglycerate mutase-like;HP_HAP_like;*
20	*Tspbctg00000007G00099420.1*	*tr|A0A0B8N0F6|A0A0B8N0F6_9EURO//Purple acid phosphatase {ECO:0000256|RuleBase:RU361203} EC=3.1.3.2 {ECO:0000256|RuleBase:RU361203} OS=Talaromyces cellulolyticus ORFNames=TCE0_011r00501 {ECO:0000313|EMBL:GAM33539.1} PE=3;*<br>*GO:0003993//acid phosphatase activity`molecular_function;*<br>*PF16656.8//Pur_ac_phosph_N`Purple acid Phosphatase, N-terminal domain;PF00149.31//Metallophos`Calcineurin-like phosphoesterase;PF14008.9//Metallophos_C`Iron/zinc purple acid phosphatase-like protein C;*<br>*InP/Purple acid Phosphatase, N-terminal domain;Purple acid phosphatase;Metallo-dependent phosphatases;Calcineurin-like phosphoesterase;Purple acid phosphatase, N-terminal domain;Iron/zinc purple acid phosphatase-like protein C; MPP_PAPs;Acid phosphatase related;*

21	Tspbctg00000006G00087650.1	InP/Phosphoglycerate mutase-like;HP_HAP_like;Histidine acid phosphatases phosphohistidine signature.;3-Phytase A;Acid_Ptase;Histidine acid phosphatases active site signature.;Histidine phosphatase superfamily (branch 2);Multiple inositol polyphosphate phosphatase-related;
22	Tspbctg00000002G00044240.1	Ref/XP_002150342.1//acid phosphatase, putative;
23	Tspbctg00000000G00005980.1	InP/Histidine phosphatase superfamily (branch 2);Phosphoglycerate mutase-like;Acid phosphatase-related;
24	Tspbctg00000004G00073010.1	InP/Acid phosphatase-related;Phosphoglycerate mutase-like;
25	Tspbctg00000003G00050000.1	PF12689.10//Acid_PPase`Acid Phosphatase;<br>InP/MDP-1: magnesium-dependent phosphatase-1;C1.5.2: MDP Like;HAD-SF-IIIC: HAD phosphatase, family IIIC;HAD_MDP-1_like;HAD-like;Acid Phosphatase;Magnesium-dependent phosphatase 1 MDP1;
26	Tspbctg00000000G00018180.1	InP/HP_PGM_like;Histidine phosphatase superfamily (branch 1);PGAM_5;Broad-range acid phosphatase det1;Phosphoglycerate mutase-like;
27	Tspbctg00000004G00063330.1	InP/acid_phosph_2;PAP2_containing_1_like;PAP2 Domain protein (AFU_ORTHOLOGUE AFUA_1G09730);PAP2 superfamily;Lipid phosphate phosphatase;Phosphatidic acid phosphatase type 2/haloperoxidase;Acid phosphatase/Vanadium-dependent haloperoxidase;
28	Tspbctg00000005G00084190.1	InP/acid_phosph_2;PAP2_containing_1_like;Phosphatidic acid phosphatase type 2/haloperoxidase;PAP2 Domain protein (AFU_ORTHOLOGUE AFUA_1G09730);Lipid phosphate phosphatase;PAP2 superfamily;Acid phosphatase/Vanadium-dependent haloperoxidase;
29	Tspbctg00000000G00012390.1	tr|A0A2H3IL55|A0A2H3IL55_9EURO//Phosphatidic acid phosphatase type 2/haloperoxidase {ECO:0000313|EMBL:PCH05612.1} OS=Penicillium sp. 'occitanis' ORFNames=PENO1_021520 {ECO:0000313|EMBL:PCH05612.1} PE=4;<br>InP/Acid phosphatase/Vanadium-dependent haloperoxidase;PAP2_Aur1_like;Phosphatidic acid phosphatase type 2/haloperoxidase;acid_phosph_2; PAP2 superfamily
**Alkaline phosphatase**		
1	Tspbctg00000002G00043660.1	K01077//E3.1.3.1, phoA, phoB`alkaline phosphatase [EC:3.1.3.1];<br>tr|A0A2H3IR64|A0A2H3IR64_9EURO//Alkaline phosphatase {ECO:0000256|ARBA:ARBA00012647, ECO:0000256|RuleBase:RU003947} EC=3.1.3.1 {ECO:0000256|ARBA:ARBA00012647, ECO:0000256|RuleBase:RU003947} OS=Penicillium sp. 'occitanis' ORFNames=PENO1_008360 {ECO:0000313|EMBL:PCH08166.1} PE=3;<br>GO:0004035//alkaline phosphatase activity`molecular_function;<br>PF00245.23//Alk_phosphatase`Alkaline phosphatase;<br>InP/Alkaline phosphatase active site.;Alkaline phosphatase;Alkaline phosphatase-related;Alkaline phosphatase signature;ALP;alk_phosph_2;Alkaline Phosphatase, subunit A;Alkaline phosphatase-like;

2	*Tspbctg00000002G00044030.1*	*InP/Non-specific phospholipase c4-related;Phosphatase, putative (AFU_ORTHOLOGUE AFUA_4G03660)-related;Phosphoesterase family;Alkaline Phosphatase, subunit A;*
3	*Tspbctg00000002G00041030.1*	*InP/Acid phosphatase phoa (AFU_ORTHOLOGUE AFUA_1G03570);Alkaline Phosphatase, subunit A;Phosphoesterase family;Non-specific phospholipase C4-related;*
4	*Tspbctg00000000G00019970.1*	*InP/Alkaline Phosphatase, subunit A;Type I phosphodiesterase / nucleotide pyrophosphatase;Alkaline phosphatase-like;*
5	*Tspbctg00000005G00074090.1*	*InP/Phosphoesterase family;phosphatase, putative (AFU_ORTHOLOGUE AFUA_4G03660)-related;non-specific phospholipase C4-related;Alkaline Phosphatase, subunit A;*
6	*Tspbctg00000004G00066990.1*	*InP/Alkaline phosphatase-like;Alkaline Phosphatase, subunit A;*
7	*Tspbctg00000000G00004850.1*	*tr|A0A2H3I075|A0A2H3I075_9EURO//Alkaline phosphatase-like, alpha/beta/alpha {ECO:0000313|EMBL:PCG90427.1} OS=Penicillium sp. 'occitanis' ORFNames=PENO1_099260 {ECO:0000313|EMBL:PCG90427.1} PE=3;<br>InP/Alkaline Phosphatase, subunit A; Alkaline phosphatase-like;*
8	*Tspbctg00000003G00054720.1*	*InP/Domain 2, Phosphonoacetate Hydrolase;HD domain;Alkaline phosphatase-like;Type I phosphodiesterase/nucleotide pyrophosphatase;Enpp;HD-domain/PDEase-like;Alkaline Phosphatase, subunit A;*
9	*Tspbctg00000003G00052810.1*	*tr|A0A2H3IFW0|A0A2H3IFW0_9EURO//Alkaline phosphatase-like, alpha/beta/alpha {ECO:0000313|EMBL:PCH00785.1} OS=Penicillium sp. 'occitanis' ORFNames=PENO1_046440 {ECO:0000313|EMBL:PCH00785.1} PE=3;<br>InP/Alkaline phosphatase-like;Alkaline Phosphatase, subunit A;*
10	*Tspbctg00000003G00057390.1*	*InP/Alkaline Phosphatase, subunit A;Alkaline phosphatase-like;*
11	*Tspbctg00000003G00055190.1*	*K01113//phoD`alkaline phosphatase D [EC:3.1.3.1];<br>tr|A0A2H3IPB5|A0A2H3IPB5_9EURO//Alkaline phosphatase D-related {ECO:0000313|EMBL:PCH07789.1} OS=Penicillium sp. 'occitanis' ORFNames=PENO1_009740 {ECO:0000313|EMBL:PCH07789.1} PE=4;<br>InP/MPP_PhoD;Metallo-dependent phosphatases;Alkaline phosphatase family protein (AFU_ORTHOLOGUE AFUA_5G03860);PhoD-like phosphatase;phosphatase, putative (AFU_ORTHOLOGUE AFUA_6G08710)-related;*
12	*Tspbctg00000003G00057840.1*	*InP/Alkaline Phosphatase, subunit A;Alkaline Phosphatase, subunit A;Phosphoesterase family;Non-specific phospholipase C4-related;*
13	*Tspbctg00000007G00099440.1*	*InP/Phosphoesterase family;Non-specific phospholipase C4-related;Alkaline Phosphatase, subunit A;*
14	*Tspbctg00000005G00080830.1*	*InP/Phosphoesterase family;Non-specific phospholipase C4-related;Specific phospholipase C, putative-related;Alkaline Phosphatase, subunit A;*
15	*Tspbctg00000005G00077980.1*	*InP/Type I phosphodiesterase/nucleotide pyrophosphatase;Alkaline phosphatase-like;Alkaline Phosphatase, subunit A;*
16	*Tspbctg00000004G00065270.1*	*tr|A0A2H3IJ01|A0A2H3IJ01_9EURO//Alkaline phosphatase-like, alpha/beta/alpha {ECO:0000313|EMBL:PCH03349.1} OS=Penicillium sp. 'occitanis' ORFNames=PENO1_032960 {ECO:0000313|EMBL:PCH03349.1} PE=4;<br>InP/Alkaline phosphatase-like;Alkaline Phosphatase, subunit A;*

17	Tspbctg00000005G00082740.1	InP/Alkaline Phosphatase, subunit A;Alkaline phosphatase-like;
18	Tspbctg00000001G00035860.1	InP/Phosphoesterase superfamily protein (AFU_ORTHOLOGUE AFUA_1G17590);Non-specific phospholipase C4-RELATED;Phosphoesterase family;Alkaline Phosphatase, subunit A;
19	Tspbctg00000003G00052820.1	InP/Alkaline Phosphatase, subunit A;Alkaline phosphatase-like;
20	Tspbctg00000006G00092560.1	InP/Alkaline phosphatase-like;Sulfatase;Alkaline Phosphatase, subunit A;
21	Tspbctg00000007G00094750.1	InP/Alkaline Phosphatase, subunit A;Alkaline phosphatase-like;
22	Tspbctg00000002G00042390.1	Nr/PCG95435.1//Alkaline phosphatase-like, alpha/beta/alpha;<br>tr|A0A2H3I6J1|A0A2H3I6J1_9EURO//Alkaline phosphatase-like, alpha/beta/alpha {ECO:0000313|EMBL:PCG95435.1} OS=Penicillium sp. 'occitanis' ORFNames=PENO1_073560 {ECO:0000313|EMBL:PCG95435.1} PE=4;<br>InP/Alkaline phosphatase-like;Alkaline Phosphatase, subunit A;
23	Tspbctg00000004G00064420.1	InP/Alkaline phosphatase-like;Alkaline Phosphatase, subunit A;
24	Tspbctg00000003G00052790.1	InP/Alkaline phosphatase-like;Alkaline Phosphatase, subunit A;
25	Tspbctg00000001G00024730.1	InP/Alkaline phosphatase-like;Alkaline Phosphatase, subunit A;
26	Tspbctg00000007G00100250.1	InP/Alkaline phosphatase-like;Alkaline Phosphatase, subunit A;
27	Tspbctg00000005G00081550.1	tr|A0A2H3J1W9|A0A2H3J1W9_9EURO//Alkaline phosphatase-like, alpha/beta/alpha {ECO:0000313|EMBL:PCH05386.1} OS=Penicillium sp. 'occitanis' ORFNames=PENO1_022730 {ECO:0000313|EMBL:PCH05386.1} PE=4;<br>InP/Alkaline phosphatase-like; Ectonucleotide pyrophosphatase/phosphodiesterase;Type I phosphodiesterase/nucleotide pyrophosphatase;Alkaline Phosphatase, subunit A; Ectonucleotide pyrophosphatase/phosphodiesterase family member 5;
28	Tspbctg00000004G00065580.1	InP/Alkaline Phosphatase, subunit A;Alkaline phosphatase-like
**Phytase**		
1	Tspbctg00000002G00042420.1	K01083//E3.1.3.8`3-phytase [EC:3.1.3.8];<br>tr|A0A6V8HBC0|A0A6V8HBC0_9EURO//3-phytase {ECO:0000256|ARBA:ARBA00012632} EC=3.1.3.8 {ECO:0000256|ARBA:ARBA00012632} OS=Talaromyces cellulolyticus ORFNames=TCE0_033f09100 {ECO:0000313|EMBL:GAM38406.1} PE=3;<br>GO:0016158//3-phytase activity`molecular_function;<br>InP/3-PHYTASE B;
2	Tspbctg00000002G00047930.1	tr|A0A2H3HYT1|A0A2H3HYT1_9EURO//3-phytase {ECO:0000256|ARBA:ARBA00012632} EC=3.1.3.8 {ECO:0000256|ARBA:ARBA00012632} OS=Penicillium sp. 'occitanis' ORFNames=PENO1_104930 {ECO:0000313|EMBL:PCG89387.1} PE=3;<br>GO:0016158//3-phytase activity`molecular_function;
3	Tspbctg00000007G00092920.1	K01083//E3.1.3.8`3-phytase [EC:3.1.3.8];

<table>
<tr><td></td><td></td><td>Nr/KAF3402268.1//3-phytase A;
tr|A0A2H3IQ07|A0A2H3IQ07_9EURO//3-phytase {ECO:0000256|ARBA:ARBA00012632} EC=3.1.3.8 {ECO:0000256|ARBA:ARBA00012632} OS=Penicillium sp. 'occitanis' ORFNames=PENO1_014230 {ECO:0000313|EMBL:PCH06943.1} PE=3;
GO:0016158//3-phytase activity`molecular_function;
InP/3-PHYTASE B;</td></tr>
<tr><td>4</td><td>Tspbctg00000007G00093540.1</td><td>Nr/KAF3402518.1//3-phytase B;
tr|B6QH60|B6QH60_TALMQ//3-phytase {ECO:0000256|ARBA:ARBA00012632} EC=3.1.3.8 {ECO:0000256|ARBA:ARBA00012632} OS=Talaromyces marneffei (strain ATCC 18224 / CBS 334.59 / QM 7333) (Penicillium marneffei) ORFNames=PMAA_093240 {ECO:0000313|EMBL:EEA22705.1} PE=3;
GO:0016158//3-phytase activity`molecular_function;</td></tr>
<tr><td>5</td><td>Tspbctg00000006G00087650.1</td><td>Nr/KAF3397168.1//3-phytase A;
tr|A0A2H3IDY7|A0A2H3IDY7_9EURO//3-phytase {ECO:0000256|ARBA:ARBA00012632} EC=3.1.3.8 {ECO:0000256|ARBA:ARBA00012632} OS=Penicillium sp. 'occitanis' ORFNames=PENO1_073200 {ECO:0000313|EMBL:PCG95521.1} PE=3;
GO:0016158//3-phytase activity`molecular_function;
InP/3-PHYTASE A;</td></tr>
<tr><td>6</td><td>Tspbctg00000000G00001750.1</td><td>Nr/KAF3401232.1//3-phytase B</td></tr>
<tr><td>7</td><td>Tspbctg00000004G00062410.1</td><td>tr|A0A6V8H8R4|A0A6V8H8R4_9EURO//Phytase-like domain-containing protein {ECO:0000259|Pfam:PF13449} OS=Talaromyces cellulolyticus ORFNames=TCE0_022r06964 {ECO:0000313|EMBL:GAM37225.1} PE=4;
PF13449.9//Phytase-like`Esterase-like activity of phytase
InP/Esterase-like activity of phytase;</td></tr>
<tr><td colspan="3">Phosphonatase</td></tr>
<tr><td>1</td><td>Tspbctg00000004G00068660.1</td><td>TIGR03351.1//PhnX-like`JCVI: phosphonatase-like hydrolase;</td></tr>
<tr><td>2</td><td>Tspbctg00000003G00055900.1</td><td>TIGR01422.1//phosphonatase`JCVI: phosphonoacetaldehyde hydrolase;</td></tr>
<tr><td>3</td><td>Tspbctg00000003G00053690.1</td><td>TIGR03351.1//PhnX-like`JCVI: phosphonatase-like hydrolase; TIGR01422.1//phosphonatase`JCVI: phosphonoacetaldehyde hydrolase;</td></tr>
<tr><td colspan="3">C-P Lyase</td></tr>
<tr><td>1</td><td>Tspbctg00000000G00001390.1</td><td>TIGR02324.1//CP_lyasePhnL`JCVI: phosphonate C-P lyase system protein PhnL;</td></tr>
<tr><td>2</td><td>Tspbctg00000003G00056240.1</td><td>TIGR02324.1//CP_lyasePhnL`JCVI: phosphonate C-P lyase system protein PhnL; TIGR02323.1//CP_lyasePhnK`JCVI: phosphonate C-P lyase system protein PhnK;</td></tr>
<tr><td>3</td><td>Tspbctg00000003G00057250.1</td><td>TIGR02323.1//CP_lyasePhnK`JCVI: phosphonate C-P lyase system protein PhnK; TIGR02324.1//CP_lyasePhnL`JCVI:</td></tr>
</table>

		phosphonate C-P lyase system protein PhnL;
4	*Tspbctg00000001G00027670.1*	*TIGR02323.1//CP_lyasePhnK`JCVI: phosphonate C-P lyase system protein PhnK; TIGR02324.1//CP_lyasePhnL`JCVI: phosphonate C-P lyase system protein PhnL;*
5	*Tspbctg00000001G00028960.1*	*TIGR02323.1//CP_lyasePhnK`JCVI: phosphonate C-P lyase system protein PhnK;*
6	*Tspbctg00000005G00077780.1*	*TIGR02324.1//CP_lyasePhnL`JCVI: phosphonate C-P lyase system protein PhnL;*
7	*Tspbctg00000007G00097290.1*	*TIGR02324.1//CP_lyasePhnL`JCVI: phosphonate C-P lyase system protein PhnL; TIGR02323.1//CP_lyasePhnK`JCVI: phosphonate C-P lyase system protein PhnK;*
8	*Tspbctg00000007G00099210.1*	*TIGR02324.1//CP_lyasePhnL`JCVI: phosphonate C-P lyase system protein PhnL;*
9	*Tspbctg00000006G00085290.1*	*TIGR02323.1//CP_lyasePhnK`JCVI: phosphonate C-P lyase system protein PhnK;*
10	*Tspbctg00000000G00011210.1*	*TIGR02323.1//CP_lyasePhnK`JCVI: phosphonate C-P lyase system protein PhnK; TIGR02324.1//CP_lyasePhnL`JCVI: phosphonate C-P lyase system protein PhnL;*
11	*Tspbctg00000001G00023290.1*	*TIGR02324.1//CP_lyasePhnL`JCVI: phosphonate C-P lyase system protein PhnL; TIGR02323.1//CP_lyasePhnK`JCVI: phosphonate C-P lyase system protein PhnK;*
12	*Tspbctg00000000G00020510.1*	*TIGR02324.1//CP_lyasePhnL`JCVI: phosphonate C-P lyase system protein PhnL;*
13	*Tspbctg00000005G00079460.1*	*TIGR02324.1//CP_lyasePhnL`JCVI: phosphonate C-P lyase system protein PhnL;*
14	*Tspbctg00000001G00031390.1*	*TIGR02323.1//CP_lyasePhnK`JCVI: phosphonate C-P lyase system protein PhnK;*
15	*Tspbctg00000002G00039210.1*	*TIGR02324.1//CP_lyasePhnL`JCVI: phosphonate C-P lyase system protein PhnL;*
16	*Tspbctg00000003G00051310.1*	*TIGR02324.1//CP_lyasePhnL`JCVI: phosphonate C-P lyase system protein PhnL;*
17	*Tspbctg00000000G00010060.1*	*TIGR02324.1//CP_lyasePhnL`JCVI: phosphonate C-P lyase system protein PhnL;*
18	*Tspbctg00000002G00041190.1*	*TIGR02324.1//CP_lyasePhnL`JCVI: phosphonate C-P lyase system protein PhnL; TIGR02323.1//CP_lyasePhnK`JCVI: phosphonate C-P lyase system protein PhnK;*
19	*Tspbctg00000006G00087240.1*	*TIGR02324.1//CP_lyasePhnL`JCVI: phosphonate C-P lyase system protein PhnL; TIGR02323.1//CP_lyasePhnK`JCVI: phosphonate C-P lyase system protein PhnK;*
20	*Tspbctg00000000G00017990.1*	*TIGR02323.1//CP_lyasePhnK`JCVI: phosphonate C-P lyase system protein PhnK; TIGR02324.1//CP_lyasePhnL`JCVI: phosphonate C-P lyase system protein PhnL;*
21	*Tspbctg00000003G00051850.1*	*TIGR02324.1//CP_lyasePhnL`JCVI: phosphonate C-P lyase system protein PhnL;*
22	*Tspbctg00000000G00018810.1*	*TIGR02324.1//CP_lyasePhnL`JCVI: phosphonate C-P lyase system protein PhnL; TIGR02323.1//CP_lyasePhnK`JCVI: phosphonate C-P lyase system protein PhnK;*
23	*Tspbctg00000004G00068440.1*	*TIGR02323.1//CP_lyasePhnK`JCVI: phosphonate C-P lyase system protein PhnK; TIGR02324.1//CP_lyasePhnL`JCVI: phosphonate C-P lyase system protein PhnL;*
24	*Tspbctg00000002G00037680.1*	*TIGR02323.1//CP_lyasePhnK`JCVI: phosphonate C-P lyase system protein PhnK;*
25	*Tspbctg00000003G00055780.1*	*TIGR02324.1//CP_lyasePhnL`JCVI: phosphonate C-P lyase system protein PhnL;*

26	*Tspbctg00000003G00061390.1*	*TIGR02323.1//CP_lyasePhnK`JCVI: phosphonate C-P lyase system protein PhnK; TIGR02324.1//CP_lyasePhnL`JCVI: phosphonate C-P lyase system protein PhnL;*
27	*Tspbctg00000006G00087510.1*	*TIGR02323.1//CP_lyasePhnK`JCVI: phosphonate C-P lyase system protein PhnK;*
28	*Tspbctg00000001G00027500.1*	*TIGR02324.1//CP_lyasePhnL`JCVI: phosphonate C-P lyase system protein PhnL;*
29	*Tspbctg00000007G00098710.1*	*TIGR02324.1//CP_lyasePhnL`JCVI: phosphonate C-P lyase system protein PhnL; TIGR02323.1//CP_lyasePhnK`JCVI: phosphonate C-P lyase system protein PhnK;*
30	*Tspbctg00000000G00005690.1*	*TIGR02323.1//CP_lyasePhnK`JCVI: phosphonate C-P lyase system protein PhnK;*
31	*Tspbctg00000000G00017240.1*	*TIGR02324.1//CP_lyasePhnL`JCVI: phosphonate C-P lyase system protein PhnL;*
32	*Tspbctg00000008G00100860.1*	*TIGR02323.1//CP_lyasePhnK`JCVI: phosphonate C-P lyase system protein PhnK*
33	*Tspbctg00000005G00081940.1*	*TIGR02324.1//CP_lyasePhnL`JCVI: phosphonate C-P lyase system protein PhnL;*
34	*Tspbctg00000006G00085170.1*	*TIGR02324.1//CP_lyasePhnL`JCVI: phosphonate C-P lyase system protein PhnL;*
35	*Tspbctg00000004G00067110.1*	*TIGR02324.1//CP_lyasePhnL`JCVI: phosphonate C-P lyase system protein PhnL;*
**Phospholipase**		
1	*Tspbctg00000002G00044030.1*	*InP/Non-specific phospholipase C4-related;*
2	*Tspbctg00000002G00042370.1*	*K13333//PLB`lysophospholipase [EC:3.1.1.5];*<br>*Nr/PCG95431.1//Acyl transferase/acyl hydrolase/lysophospholipase;*<br>*tr|A0A2H3I6U2|A0A2H3I6U2_9EURO//Lysophospholipase {ECO:0000256|ARBA:ARBA00013274, ECO:0000256|RuleBase:RU362103} EC=3.1.1.5 {ECO:0000256|ARBA:ARBA00013274, ECO:0000256|RuleBase:RU362103} OS=Penicillium sp. 'occitanis' ORFNames=PENO1_073520 {ECO:0000313|EMBL:PCG95431.1} PE=3;*<br>*GO:0004622//lysophospholipase activity`molecular_function; GO:0102545//phosphatidyl phospholipase B activity`molecular_function;*<br>*KOG1325`Lysophospholipase`Lipid transport and metabolism;*<br>*PF01735.21//PLA2_B`Lysophospholipase catalytic domain;*<br>*InP/Lysophospholipase catalytic domain;Cytosolic phospholipase A2 catalytic domain;cytosolic phospholipase A2;FabD/lysophospholipase-like;lysophospholipase;pla2_6;PLA2c domain profile.*
3	*Tspbctg00000002G00040920.1*	*K14674//TGL4`TAG lipase / steryl ester hydrolase / phospholipase A2 / LPA acyltransferase [EC:3.1.1.3 3.1.1.13 3.1.1.4 2.3.1.51];*<br>*tr|A0A6V8HBA0|A0A6V8HBA0_9EURO//Patatin-like phospholipase domain-containing protein {ECO:0000256|RuleBase:RU362055} EC=3.1.1.- {ECO:0000256|RuleBase:RU362055} OS=Talaromyces cellulolyticus ORFNames=TCE0_033f09398 {ECO:0000313|EMBL:GAM38571.1} PE=3;*<br>*PF01734.25//Patatin`Patatin-like phospholipase;*

		InP/FabD/lysophospholipase-like;neuropathy target esterase/swiss cheese d.melanogaster;Pat_TGL4-5_like;lipase 4-related;Patatin-like phospholipase (PNPLA) domain profile.;Patatin-like phospholipase;Cytosolic phospholipase A2 catalytic domain;Cytosolic phospholipase A2 catalytic domain;
4	*Tspbctg00000002G00044450.1*	*K13535//CLD1`cardiolipin-specific phospholipase [EC:3.1.1.-];*
5	*Tspbctg00000002G00041030.1*	*InP/Non-specific phospholipase c4-related;*
6	*Tspbctg00000004G00072400.1*	*InP/Phospholipase-related;*
7	*Tspbctg00000004G00071360.1*	*K18694//PGC1`phosphatidylglycerol phospholipase C [EC:3.1.4.-];*
8	*Tspbctg00000005G00074090.1*	*InP/Non-specific phospholipase c4-related;*
9	*Tspbctg00000000G00016040.1*	*K18694//PGC1`phosphatidylglycerol phospholipase C [EC:3.1.4.-];*<br>*InP/Phosphatidylinositol-specific phospholipase X-box domain profile.;*
10	*Tspbctg00000004G00066750.1*	*PF01734.25//Patatin`Patatin-like phospholipase;*<br>*InP/Pat_TGL3_like;LIPASE 3;Patatin-like phospholipase (PNPLA) domain profile.;neuropathy target esterase/swiss cheese d.melanogaster;FabD/lysophospholipase-like;Patatin-like phospholipase;Cytosolic phospholipase A2 catalytic domain;*
11	*Tspbctg00000004G00070370.1*	*PF00388.22//PI-PLC-X`Phosphatidylinositol-specific phospholipase C, X domain;*<br>*InP/Phosphatidylinositol-specific phospholipase C, X domain;Phosphatidylinositol-specific phospholipase X-box domain profile.;*
12	*Tspbctg00000006G00090480.1*	*Nr/PCG94490.1//Acyl transferase/acyl hydrolase/lysophospholipase;*<br>*InP/FabD/lysophospholipase-like;*
13	*Tspbctg00000006G00087920.1*	*KOG2551`Phospholipase/carboxyhydrolase`Amino acid transport and metabolism*
14	*Tspbctg00000006G00090070.1*	*K01115//PLD1_2`phospholipase D1/2 [EC:3.1.4.4];*<br>*Nr/KAF3397461.1//Phospholipase D1;*<br>*tr|A0A6V8HPK9|A0A6V8HPK9_9EURO//Phospholipase {ECO:0000256|PIRNR:PIRNR009376} EC=3.1.4.4 {ECO:0000256|PIRNR:PIRNR009376} OS=Talaromyces cellulolyticus ORFNames=TCE0_060f18544 {ECO:0000313|EMBL:GAM43603.1} PE=3;*<br>*GO:0070290//N-acylphosphatidylethanolamine-specific phospholipase D activity`molecular_function;GO:0004630//phospholipase D activity`molecular_function;*<br>*PF00614.25//PLDc`Phospholipase D Active site motif;PF13091.9//PLDc_2`PLD-like domain;*<br>*InP/PLD_euk;pld_4;PX domain;PLDc_vPLD1_2_yPLD_like_2;PX_2;PX domain profile.;Phospholipase d;Phospholipase D/nuclease;PH_PLD;Phospholipase D phosphodiesterase active site profile.;PLD-like domain;PLDc_vPLD1_2_yPLD_like_1;Phospholipase D Active site motif;Phospholipase d1;*
15	*Tspbctg00000006G00090470.1*	*InP/FabD/lysophospholipase-like;*

16	Tspbctg00000000G00000350.1	K01115//PLD1_2`phospholipase D1/2 [EC:3.1.4.4];<br>Nr/PCH05249.1//Phospholipase D family;<br>tr|A0A2H3J3Z8|A0A2H3J3Z8_9EURO//Phospholipase D {ECO:0000256|ARBA:ARBA00012027} EC=3.1.4.4 {ECO:0000256|ARBA:ARBA00012027} OS=Penicillium sp. 'occitanis' ORFNames=PENO1_023500 {ECO:0000313|EMBL:PCH05249.1} PE=4;<br>GO:0004630//phospholipase D activity`molecular_function;GO:0070290//N-acylphosphatidylethanolamine-specific phospholipase D activity`molecular_function;<br>InP/Phospholipase D/nuclease;phospholipase;Phospholipase D phosphodiesterase active site profile.;Phospholipase D;Phospholipase D Active site motif; PLDc_vPLD1_2_yPLD_like_1;PLDc_vPLD1_2_yPLD_like_2;pld_4;<br>Ref/XP_002145583.1//phospholipase D (PLD), putative;
17	Tspbctg00000006G00091460.1	Nr/PCH02215.1//Acyl transferase/acyl hydrolase/lysophospholipase;<br>tr|A0A2H3IG86|A0A2H3IG86_9EURO//Lysophospholipase NTE1 {ECO:0000256|RuleBase:RU362043} EC=3.1.1.5 {ECO:0000256|RuleBase:RU362043} Intracellular phospholipase B {ECO:0000256|RuleBase:RU362043} OS=Penicillium sp. 'occitanis' ORFNames=PENO1_039030 {ECO:0000313|EMBL:PCH02215.1} PE=3;<br>GO:0004622//lysophospholipase activity`molecular_function;GO:0102545//phosphatidyl phospholipase B activity`molecular_function;<br>InP/FabD/lysophospholipase-like;Patatin-like phospholipase;Cytosolic phospholipase A2 catalytic domain;Patatin-like phospholipase (PNPLA) domain profile.;
18	Tspbctg00000003G00051450.1	K14018//PLAA, DOA1, UFD3`phospholipase A-2-activating protein;<br>KOG0301`Phospholipase A2-activating protein (contains WD40 repeats)`Lipid transport and metabolism;<br>InP/Phospholipase a2-activating protein;
19	Tspbctg00000000G00009860.1	InP/FabD/lysophospholipase-like;
20	Tspbctg00000003G00056760.1	InP/pld_4;PLD_PSS;Phospholipase D/nuclease;Phospholipase D phosphodiesterase active site profile.;PLDc_PGS1_euk_2;PLDc_PGS1_euk_1;PLD-like domain;
21	Tspbctg00000003G00057840.1	K01114//plc`phospholipase C [EC:3.1.4.3];<br>InP/Non-specific phospholipase c4-related;<br>TIGR03396.1//PC_PLC`JCVI: phospholipase C, phosphocholine-specific;
22	Tspbctg00000001G00029500.1	InP/FabD/lysophospholipase-like;
23	Tspbctg00000001G00028560.1	K05857//PLCD`phosphatidylinositol phospholipase C, delta [EC:3.1.4.11];<br>tr|A0A2H3IJ35|A0A2H3IJ35_9EURO//Phosphoinositide phospholipase C {ECO:0000256|ARBA:ARBA00012368, ECO:0000256|RuleBase:RU361133} EC=3.1.4.11 {ECO:0000256|ARBA:ARBA00012368, ECO:0000256|RuleBase:RU361133} OS=Penicillium sp. 'occitanis' ORFNames=PENO1_034600

		{ECO:0000313|EMBL:PCH03010.1} PE=4;<br>GO:0004435//phosphatidylinositol phospholipase C activity`molecular_function;<br>KOG0169`Phosphoinositide-specific phospholipase C`Signal transduction mechanisms;<br>PF00388.22//PI-PLC-X`Phosphatidylinositol-specific phospholipase C, X domain;PF00387.22//PI-PLC-Y`Phosphatidylinositol-specific phospholipase C, Y domain;<br>InP/Phosphatidylinositol (PI) phosphodiesterase;Phosphatidylinositol-specific phospholipase C, X domain;Phosphatidylinositol-specific phospholipase X-box domain profile.;Phosphoinositide phospholipase c;PH domain-like;Phosphoinositide-specific phospholipase c family protein;PI-PLC1c_yeast;PH_PLC_fungal;Phosphatidylinositol-specific phospholipase C, Y domain;C2_3c;Phosphatidylinositol-specific phospholipase Y-box domain profile.;plcy_3;Phospholipase C signature;
24	Tspbctg00000001G00026050.1	K13333//PLB`lysophospholipase [EC:3.1.1.5];<br>Nr/GAM41995.1//phospholipase;<br>tr|A0A0B8MY70|A0A0B8MY70_9EURO//Lysophospholipase {ECO:0000256|ARBA:ARBA00013274, ECO:0000256|RuleBase:RU362103} EC=3.1.1.5 {ECO:0000256|ARBA:ARBA00013274, ECO:0000256|RuleBase:RU362103} OS=Talaromyces cellulolyticus ORFNames=TCE0_043f15576 {ECO:0000313|EMBL:GAM41995.1} PE=3;<br>GO:0004622//lysophospholipase activity`molecular_function;GO:0102545//phosphatidyl phospholipase B activity`molecular_function;<br>KOG1325`Lysophospholipase`Lipid transport and metabolism;<br>PF01735.21//PLA2_B`Lysophospholipase catalytic domain;<br>InP/Lysophospholipase catalytic domain;FabD/lysophospholipase-like;pla2_6;PLA2c domain profile.;Cytosolic phospholipase A2 catalytic domain;Lysophospholipase;Cytosolic phospholipase A2;<br>Ref/XP_046078491.1//lysophospholipase catalytic domain-containing protein;
25	Tspbctg00000007G00099440.1	K01114//plc`phospholipase C [EC:3.1.4.3];<br>InP/Non-specific phospholipase C4-related;<br>TIGR03396.1//PC_PLC`JCVI: phospholipase C, phosphocholine-specific;
26	Tspbctg00000005G00080830.1	K01114//plc`phospholipase C [EC:3.1.4.3];<br>Nr/KAF3390825.1//Non-specific phospholipase C6;<br>InP/Non-specific phospholipase C4-related;Specific phospholipase c, Putative-related;<br>TIGR03396.1//PC_PLC`JCVI: phospholipase C, phosphocholine-specific;
27	Tspbctg00000001G00034340.1	InP/Phosphatidylinositol-specific phospholipase X-box domain profile;
28	Tspbctg00000005G00084010.1	K01115//PLD1_2`phospholipase D1/2 [EC:3.1.4.4];

		Nr/PCG90810.1//Phospholipase D family;<br>tr|A0A2H3I236|A0A2H3I236_9EURO//Phospholipase D {ECO:0000256|ARBA:ARBA00012027} EC=3.1.4.4 {ECO:0000256|ARBA:ARBA00012027} OS=Penicillium sp. 'occitanis' ORFNames=PENO1_097190 {ECO:0000313|EMBL:PCG90810.1} PE=4;<br>GO:0004630//phospholipase D activity`molecular_function;GO:0070290//N-acylphosphatidylethanolamine-specific phospholipase D activity`molecular_function;<br>PF00614.25//PLDc`Phospholipase D Active site motif;<br>InP/Phospholipase D Active site motif;PLD_euk;Phospholipase D phosphodiesterase active site profile.;PLDc_vPLD1_2_yPLD_like_2;PLDc_vPLD1_2_yPLD_like_1;Phospholipase D phosphodiesterase active site profile.;Phospholipase;Phospholipase D/nuclease;Phospholipase D;pld_4;PLD-like domain;
29	*Tspbctg00000005G00083540.1*	K16342//PLA2G4, CPLA2`cytosolic phospholipase A2 [EC:3.1.1.4];<br>tr|A0A6V8HBA2|A0A6V8HBA2_9EURO//Lysophospholipase {ECO:0000256|ARBA:ARBA00013274, ECO:0000256|RuleBase:RU362103} EC=3.1.1.5 {ECO:0000256|ARBA:ARBA00013274, ECO:0000256|RuleBase:RU362103} OS=Talaromyces cellulolyticus ORFNames=TCE0_024f07560 {ECO:0000313|EMBL:GAM37549.1} PE=3;<br>GO:0004622//lysophospholipase activity`molecular_function;GO:0102545//phosphatidyl phospholipase B activity`molecular_function;<br>KOG1325`Lysophospholipase`Lipid transport and metabolism;<br>PF01735.21//PLA2_B`Lysophospholipase catalytic domain;<br>InP/PLA2c domain profile.;Cytosolic phospholipase A2;cPLA2_like; Lysophospholipase;pla2_6;Lysophospholipase catalytic domain;FabD/lysophospholipase-like;Cytosolic phospholipase A2 catalytic domain;
30	*Tspbctg00000005G00082560.1*	InP/FabD/lysophospholipase-like;
31	*Tspbctg00000005G00082570.1*	tr|A0A2H3IIM8|A0A2H3IIM8_9EURO//Acyl transferase/acyl hydrolase/lysophospholipase {ECO:0000313|EMBL:PCH03972.1} OS=Penicillium sp. 'occitanis' ORFNames=PENO1_029740 {ECO:0000313|EMBL:PCH03972.1} PE=3;<br>InP/FabD/lysophospholipase-like;
32	*Tspbctg00000001G00035860.1*	K01114//plc`phospholipase C [EC:3.1.4.3];<br>Nr/KAF3394773.1//Non-specific phospholipase C6;<br>InP/Non-specific phospholipase C4-related;
33	*Tspbctg00000002G00041220.1*	Nr/KAF3399312.1//Calcium-independent phospholipase A2-gamma;<br>KOG4231`Intracellular membrane-bound Ca2+-independent phospholipase A2`Lipid transport and metabolism;<br>PF01734.25//Patatin`Patatin-like phospholipase;

		InP/Cytosolic phospholipase A2 catalytic domain;Calcium-independent phospholipase A2-gamma;Patatin-like phospholipase;FabD/lysophospholipase-like;
34	*Tspbctg00000005G00079910.1*	*InP/FabD/lysophospholipase-like;*
35	*Tspbctg00000005G00076220.1*	*InP/FabD/lysophospholipase-like;*
36	*Tspbctg00000000G00002140.1*	*KOG2308`Phosphatidic acid-preferring phospholipase A1, contains DDHD domain`Lipid transport and metabolism;KOG2308`Phosphatidic acid-preferring phospholipase A1, contains DDHD domain`Intracellular trafficking, secretion, and vesicular transport;*<br>*InP/DDHD_2a;DDHD domain;Phospholipase C1020.13C-related;DDHD domain profile.;PA-PL1 Phospholipase family;*
37	*Tspbctg00000005G00082590.1*	*InP/FabD/lysophospholipase-like;*
38	*Tspbctg00000006G00086140.1*	*InP/Phospholipase-related;*
39	*Tspbctg00000003G00053220.1*	*PF02230.19//Abhydrolase_2`Phospholipase/Carboxylesterase;*<br>*InP/Phospholipase/Carboxylesterase;Lysophospholipase-related;*
40	*Tspbctg00000001G00033970.1*	*KOG2308`Phosphatidic acid-preferring phospholipase A1, contains DDHD domain`Lipid transport and metabolism;KOG2308`Phosphatidic acid-preferring phospholipase A1, contains DDHD domain`Intracellular trafficking, secretion, and vesicular transport;*
41	*Tspbctg00000003G00061570.1*	*InP/Phospholipase C/P1 nuclease;*
42	*Tspbctg00000004G00063210.1*	*InP/FabD/lysophospholipase-like;*
43	*Tspbctg00000007G00093250.1*	*KOG2551`Phospholipase/carboxyhydrolase`Amino acid transport and metabolism;*
44	*Tspbctg00000005G00083770.1*	*KOG2551`Phospholipase/carboxyhydrolase`Amino acid transport and metabolism;*
45	*Tspbctg00000001G00035490.1*	*PF02230.19//Abhydrolase_2`Phospholipase/Carboxylesterase;*<br>*InP/Phospholipase/Carboxylesterase;Lysophospholipase-related;*
46	*Tspbctg00000004G00073610.1*	*KOG2551`Phospholipase/carboxyhydrolase`Amino acid transport and metabolism;*
47	*Tspbctg00000004G00073600.1*	*PCG90706.1//Acyl transferase/acyl hydrolase/lysophospholipase;*<br>*tr|A0A2H3I7B4|A0A2H3I7B4_9EURO//Acyl transferase/acyl hydrolase/lysophospholipase {ECO:0000313|EMBL:PCG90706.1} OS=Penicillium sp. 'occitanis' ORFNames=PENO1_097720 {ECO:0000313|EMBL:PCG90706.1} PE=4;*
48	*Tspbctg00000003G00054170.1*	*tr|A0A2H3J3V3|A0A2H3J3V3_9EURO//Acyl transferase/acyl hydrolase/lysophospholipase {ECO:0000313|EMBL:PCH06684.1} OS=Penicillium sp. 'occitanis' ORFNames=PENO1_015750 {ECO:0000313|EMBL:PCH06684.1} PE=4;*<br>*InP/FabD/lysophospholipase-like;*
49	*Tspbctg00000003G00055850.1*	*tr|A0A510NV38|A0A510NV38_9EURO//Phospholipase/carboxylesterase superfamily protein {ECO:0000313|EMBL:GAM36117.1} OS=Talaromyces cellulolyticus ORFNames=TCE0_018r04956*

<table>
<tr><td></td><td></td><td>{ECO:0000313|EMBL:GAM36117.1} PE=4;
PF02230.19//Abhydrolase_2`Phospholipase/Carboxylesterase;
InP/Phospholipase/Carboxylesterase;Lysophospholipase-related;Phospholipase/carboxylesterase superfamily (AFU_ORTHOLOGUE AFUA_5G09340);</td></tr>
<tr><td>50</td><td>Tspbctg00000005G00082330.1</td><td>InP/FabD/lysophospholipase-like;</td></tr>
<tr><td>51</td><td>Tspbctg00000005G00082320.1</td><td>KOG2551`Phospholipase/carboxyhydrolase`Amino acid transport and metabolism;</td></tr>
<tr><td>52</td><td>Tspbctg00000004G00071340.1</td><td>KOG2551`Phospholipase/carboxyhydrolase`Amino acid transport and metabolism;
PF02230.19//Abhydrolase_2`Phospholipase/Carboxylesterase;</td></tr>
<tr><td>53</td><td>Tspbctg00000006G00092230.1</td><td>PF02230.19//Abhydrolase_2`Phospholipase/Carboxylesterase;
InP/Lysophospholipase-related;Phospholipase/Carboxylesterase;</td></tr>
<tr><td>54</td><td>Tspbctg00000001G00030930.1</td><td>InP/FabD/lysophospholipase-like;</td></tr>
<tr><td>55</td><td>Tspbctg00000006G00086000.1</td><td>GO:0070290//N-acylphosphatidylethanolamine-specific phospholipase D activity`molecular_function;
InP/N-ACYL-Phosphatidylethanolamine-hydrolyzing phospholipase D;</td></tr>
<tr><td>56</td><td>Tspbctg00000002G00048200.1</td><td>PF02230.19//Abhydrolase_2`Phospholipase/Carboxylesterase;
InP/Phospholipase/Carboxylesterase;Lysophospholipase-related;</td></tr>
<tr><td>57</td><td>Tspbctg00000000G00022530.1</td><td>InP/FabD/lysophospholipase-like;</td></tr>
<tr><td>58</td><td>Tspbctg00000001G00033450.1</td><td>Nr/KAF3394958.1//Phospholipase A I;
tr|A0A2H3ICC0|A0A2H3ICC0_9EURO//Patatin/Phospholipase A2-related {ECO:0000313|EMBL:PCG98303.1} OS=Penicillium sp. 'occitanis' ORFNames=PENO1_058840 {ECO:0000313|EMBL:PCG98303.1} PE=4;
KOG4231`Intracellular membrane-bound Ca2+-independent phospholipase A2`Lipid transport and metabolism;
PF01734.25//Patatin`Patatin-like phospholipase;
InP/Patatin-like phospholipase (PNPLA) domain profile.;Calcium-independent phospholipase a2-gamma;Pat17_PNPLA8_PNPLA9_like3;Patatin-like phospholipase;FabD/lysophospholipase-like;Cytosolic phospholipase A2 catalytic domain;</td></tr>
<tr><td>59</td><td>Tspbctg00000005G00082370.1</td><td>Nr/PCH03991.1//Acyl transferase/acyl hydrolase/lysophospholipase;
tr|A0A2H3IJZ1|A0A2H3IJZ1_9EURO//Acyl transferase/acyl hydrolase/lysophospholipase {ECO:0000313|EMBL:PCH03991.1} OS=Penicillium sp. 'occitanis' ORFNames=PENO1_029930 {ECO:0000313|EMBL:PCH03991.1} PE=4;
InP/FabD/lysophospholipase-like;</td></tr>
<tr><td>60</td><td>Tspbctg00000000G00017360.1</td><td>InP/Phospholipase C/P1 nuclease;</td></tr>
<tr><td>61</td><td>Tspbctg00000001G00026850.1</td><td>InP/N-ACYL-Phosphatidylethanolamine-hydrolyzing phospholipase D;</td></tr>
<tr><td>62</td><td>Tspbctg00000004G00070120.1</td><td>InP/Phospholipase-related;</td></tr>
</table>

<table>
<tr><td>63</td><td>Tspbctg00000004G00071350.1</td><td>Nr/KAF3393684.1//N-acyl-phosphatidylethanolamine-hydrolyzing phospholipase D;
GO:0070290//N-acylphosphatidylethanolamine-specific phospholipase D activity`molecular_function;
InP/N-ACYL-Phosphatidylethanolamine-hydrolyzing phospholipase D;NAPE-PLD;</td></tr>
<tr><td>64</td><td>Tspbctg00000000G00016310.1</td><td>KOG2551`Phospholipase/carboxyhydrolase`Amino acid transport and metabolism;</td></tr>
<tr><td>65</td><td>Tspbctg00000004G00073510.1</td><td>InP/FabD/lysophospholipase-like;</td></tr>
<tr><td>66</td><td>Tspbctg00000003G00054670.1</td><td>tr|A0A2H3IPX7|A0A2H3IPX7_9EURO//Acyl transferase/acyl hydrolase/lysophospholipase
{ECO:0000313|EMBL:PCG95458.1} OS=Penicillium sp. 'occitanis' ORFNames=PENO1_073380
{ECO:0000313|EMBL:PCG95458.1} PE=4;
InP/FabD/lysophospholipase-like;</td></tr>
<tr><td>67</td><td>Tspbctg00000003G00049270.1</td><td>InP/Phospholipase D/nuclease;Phospholipase D phosphodiesterase active site profile.;</td></tr>
<tr><td>68</td><td>Tspbctg00000006G00090900.1</td><td>InP/FabD/lysophospholipase-like;</td></tr>
<tr><td>69</td><td>Tspbctg00000007G00093590.1</td><td>tr|A0A2H3I6K9|A0A2H3I6K9_9EURO//Phospholipase/carboxylesterase/thioesterase
{ECO:0000313|EMBL:PCG95280.1} OS=Penicillium sp. 'occitanis' ORFNames=PENO1_074320
{ECO:0000313|EMBL:PCG95280.1} PE=4;
PF02230.19//Abhydrolase_2`Phospholipase/Carboxylesterase;
InP/Phospholipase/Carboxylesterase;Lysophospholipase-related;</td></tr>
<tr><td>70</td><td>Tspbctg00000005G00082540.1</td><td>KOG2551`Phospholipase/carboxyhydrolase`Amino acid transport and metabolism;</td></tr>
<tr><td>71</td><td>Tspbctg00000001G00030920.1</td><td>tr|A0A2H3IQH8|A0A2H3IQH8_9EURO//Acyl transferase/acyl hydrolase/lysophospholipase
{ECO:0000313|EMBL:PCG99293.1} OS=Penicillium sp. 'occitanis' ORFNames=PENO1_053750
{ECO:0000313|EMBL:PCG99293.1} PE=4;
InP/FabD/lysophospholipase-like;</td></tr>
<tr><td>72</td><td>Tspbctg00000005G00082390.1</td><td>InP/Phospholipase-related;</td></tr>
<tr><td>73</td><td>Tspbctg00000000G00016580.1</td><td>InP/N-ACYL-Phosphatidylethanolamine-hydrolyzing phospholipase D;</td></tr>
<tr><td>74</td><td>Tspbctg00000005G00077480.1</td><td>InP/Phospholipase D/nuclease;Phospholipase D phosphodiesterase active site profile.;PLDc_Tdp1_2;</td></tr>
<tr><td>75</td><td>Tspbctg00000000G00002690.1</td><td>InP/FabD/lysophospholipase-like;</td></tr>
<tr><td>76</td><td>Tspbctg00000001G00034000.1</td><td>KOG2308`Phosphatidic acid-preferring phospholipase A1, contains DDHD domain`Lipid transport and metabolism;KOG2308`Phosphatidic acid-preferring phospholipase A1, contains DDHD domain`Intracellular trafficking, secretion, and vesicular transport;
InP/PA-PL1 Phospholipase family;LD21067P;DDHD domain profile.;DDHD_2a;DDHD domain;</td></tr>
<tr><td>77</td><td>Tspbctg00000001G00024620.1</td><td>InP/FabD/lysophospholipase-like;</td></tr>
<tr><td>78</td><td>Tspbctg00000005G00074870.1</td><td>tr|A0A2H3I8X2|A0A2H3I8X2_9EURO//Phospholipase/carboxylesterase/thioesterase
{ECO:0000313|EMBL:PCG97246.1} OS=Penicillium sp. 'occitanis' ORFNames=PENO1_064340</td></tr>
</table>

		{ECO:0000313|EMBL:PCG97246.1} PE=4;<br>PF02230.19//Abhydrolase_2`Phospholipase/Carboxylesterase;<br>InP/Phospholipase/Carboxylesterase;Lysophospholipase-related;
79	Tspbctg00000005G00080970.1	Nr/PCH08573.1//Acyl transferase/acyl hydrolase/lysophospholipase;<br>tr|A0A2H3IQF6|A0A2H3IQF6_9EURO//Acyl transferase/acyl hydrolase/lysophospholipase<br>{ECO:0000313|EMBL:PCH08573.1} OS=Penicillium sp. 'occitanis' ORFNames=PENO1_006380<br>{ECO:0000313|EMBL:PCH08573.1} PE=4;<br>InP/FabD/lysophospholipase-like;
80	Tspbctg00000001G00028890.1	InP/Regulator of phospholipase D SRF1
81	Tspbctg00000007G00093290.1	Nr/PCG93692.1//Acyl transferase/acyl hydrolase/lysophospholipase;<br>tr|A0A2H3IBE2|A0A2H3IBE2_9EURO//Acyl transferase/acyl hydrolase/lysophospholipase<br>{ECO:0000313|EMBL:PCG93692.1} OS=Penicillium sp. 'occitanis' ORFNames=PENO1_082350<br>{ECO:0000313|EMBL:PCG93692.1} PE=4;<br>InP/FabD/lysophospholipase-like;
82	Tspbctg00000000G00001520.1	InP/Thioesterase 1/protease 1/lysophospholipase L1;
83	Tspbctg00000006G00085990.1	Nr/KAF3397545.1//N-acyl-phosphatidylethanolamine-hydrolyzing phospholipase D;
84	Tspbctg00000000G00022630.1	InP/Phosphatidylinositol-specific phospholipase X-box domain profile.;
85	Tspbctg00000006G00086260.1	Nr/PCG99707.1//Acyl transferase/acyl hydrolase/lysophospholipase;<br>tr|A0A2H3IDL1|A0A2H3IDL1_9EURO//Acyl transferase/acyl hydrolase/lysophospholipase<br>{ECO:0000313|EMBL:PCG99707.1} OS=Penicillium sp. 'occitanis' ORFNames=PENO1_051860<br>{ECO:0000313|EMBL:PCG99707.1} PE=4;<br>InP/FabD/lysophospholipase-like;
86	Tspbctg00000003G00049850.1	InP/FabD/lysophospholipase-like;
87	Tspbctg00000004G00065040.1	InP/FabD/lysophospholipase-like;
88	Tspbctg00000001G00035630.1	tr|A0A2H3IKA2|A0A2H3IKA2_9EURO//Acyl transferase/acyl hydrolase/lysophospholipase<br>{ECO:0000313|EMBL:PCH05023.1} OS=Penicillium sp. 'occitanis' ORFNames=PENO1_024690<br>{ECO:0000313|EMBL:PCH05023.1} PE=4;<br>InP/FabD/lysophospholipase-like;
89	Tspbctg00000000G00017480.1	InP/Phospholipase-related;
90	Tspbctg00000002G00047440.1	InP/FabD/lysophospholipase-like;

Dolichyldiphosphatase

1	*Tspbctg00000000G00020390.1*	*K07252//DOLPP1`dolichyldiphosphatase [EC:3.6.1.43]; tr|A0A6V8HI27|A0A6V8HI27_9EURO//Dolichyldiphosphatase {ECO:0000256|RuleBase:RU367078} EC=3.6.1.43 {ECO:0000256|RuleBase:RU367078} OS=Talaromyces cellulolyticus ORFNames=TCE0_034r11736 {ECO:0000313|EMBL:GAM39855.1} PE=3； GO:0047874//Dolichyldiphosphatase activity`molecular_function; InP/Phosphatidic acid phosphatase type 2/haloperoxidase;palmitoyl-protein thioesterase/dolichyldiphosphatase 1;Acid phosphatase/Vanadium-dependent haloperoxidase;PAP2 superfamily;PAP2_dolichyldiphosphatase;Dolichyldiphosphatase 1;acid_phosph_2；*
**Pyrophosphatase**		
1	*Tspbctg00000000G00019970.1*	*InP/Type I phosphodiesterase/nucleotide pyrophosphatase;*
2	*Tspbctg00000002G00048520.1*	*K01520//dut, DUT`dUTP pyrophosphatase [EC:3.6.1.23]; tr|A0A2H3IG74|A0A2H3IG74_9EURO//Deoxyuridine 5'-triphosphate nucleotidohydrolase {ECO:0000256|RuleBase:RU367024} Short=dUTPase {ECO:0000256|RuleBase:RU367024} EC=3.6.1.23 {ECO:0000256|RuleBase:RU367024} dUTP pyrophosphatase {ECO:0000256|RuleBase:RU367024} OS=Penicillium sp. 'occitanis' ORFNames=PENO1_074670 {ECO:0000313|EMBL:PCG95182.1} PE=3;*
3	*Tspbctg00000003G00054190.1*	*GO:0016462//pyrophosphatase activity`molecular_function;*
4	*Tspbctg00000003G00054720.1*	*Nr/PCH07840.1//Type I phosphodiesterase / nucleotide pyrophosphatase/phosphate transferase; tr|A0A2H3IWS6|A0A2H3IWS6_9EURO//Type I phosphodiesterase/nucleotide pyrophosphatase/phosphate transferase {ECO:0000313|EMBL:PCH07840.1} OS=Penicillium sp. 'occitanis' ORFNames=PENO1_010250 {ECO:0000313|EMBL:PCH07840.1} PE=4; PF01663.25//Phosphodiest`Type I phosphodiesterase / nucleotide pyrophosphatase; InP/Type I phosphodiesterase / nucleotide pyrophosphatase;*
5	*Tspbctg00000003G00054320.1*	*Nr/KAF3406520.1//NADH pyrophosphatase; PF09296.14//NUDIX-like`NADH pyrophosphatase-like rudimentary NUDIX domain;PF09297.14//zf-NADH-PPase`NADH pyrophosphatase zinc ribbon domain;PF00293.31//NUDIX`NUDIX domain; InP/Nudix hydrolase, nudc subfamily;NADH pyrophosphatase-like rudimentary NUDIX domain;Nudix box signature.;Peroxisomal nadh pyrophosphatase nudt12;NUDIX domain;Nudix;NADH pyrophosphatase zinc ribbon domain;Nucleoside Triphosphate Pyrophosphohydrolase;NADH_pyrophosphatase;Nudix hydrolase domain profile.; Ref/XP_002147731.1//NADH pyrophosphatase, putative;*
6	*Tspbctg00000003G00055890.1*	*K01515//nudF`ADP-ribose pyrophosphatase [EC:3.6.1.13]; InP/ADPRase_NUDT5;Nucleoside Triphosphate Pyrophosphohydrolase;NUDIX domain;Nudix box signature.;Nudix*

<table>
<tr><td></td><td></td><td>hydrolase domain profile.;NUDIX hydrolase family signature;NUDIX hydrolase family signature;UDP/ADP-Sugar pyrophosphatase;Nudix;ADP-Sugar pyrophosphatase;</td></tr>
<tr><td>7</td><td>Tspbctg00000003G00058540.1</td><td>K01519//ITPA`inosine triphosphate pyrophosphatase [EC:3.6.1.-];
Nr/KAF3407536.1//Inosine triphosphate pyrophosphatase;
tr|A0A0B8N0Z9|A0A0B8N0Z9_9EURO//Inosine triphosphate pyrophosphatase {ECO:0000256|HAMAP-Rule:MF_03148} Short=ITPase {ECO:0000256|HAMAP-Rule:MF_03148} Short=Inosine triphosphatase {ECO:0000256|HAMAP-Rule:MF_03148} EC=3.6.1.9 {ECO:0000256|HAMAP-Rule:MF_03148} Non-canonical purine NTP pyrophosphatase {ECO:0000256|HAMAP-Rule:MF_03148} Non-standard purine NTP pyrophosphatase {ECO:0000256|HAMAP-Rule:MF_03148} Nucleoside-triphosphate diphosphatase {ECO:0000256|HAMAP-Rule:MF_03148} Nucleoside-triphosphate pyrophosphatase {ECO:0000256|HAMAP-Rule:MF_03148} Short=NTPase {ECO:0000256|HAMAP-Rule:MF_03148} OS=Talaromyces cellulolyticus ORFNames=TCE0_038r12254 {ECO:0000313|EMBL:GAM40139.1} PE=3;
GO:0035529//NADH pyrophosphatase activity`molecular_function;
InP/Inosine triphosphate pyrophosphatase/ham1 protein;Inosine triphosphate pyrophosphatase [ITPA].;ITPase-like;TIGR00042: non-canonical purine NTP pyrophosphatase, RdgB/HAM1 family;Ham1 family;HAM1;
Ref/XP_002148152.1//non-canonical purine NTP pyrophosphatase, rdgB/HAM1 family;
TIGR00042.1//TIGR00042`JCVI: RdgB/HAM1 family non-canonical purine NTP pyrophosphatase;</td></tr>
<tr><td>8</td><td>Tspbctg00000007G00099450.1</td><td>GO:0016462//pyrophosphatase activity`molecular_function;
InP/GMP synthetase ATP pyrophosphatase (GMPS ATP-PPase) domain profile.;</td></tr>
<tr><td>9</td><td>Tspbctg00000005G00075900.1</td><td>K01507//ppa`inorganic pyrophosphatase [EC:3.6.1.1];<br>Nr/KAF3387909.1//Inorganic pyrophosphatase;<br>PF00719.22//Pyrophosphatase`Inorganic pyrophosphatase;
InP/Inorganic pyrophosphatase;Inorganic pyrophosphatase signature.;pyrophosphatase;</td></tr>
<tr><td>10</td><td>Tspbctg00000005G00077980.1</td><td>InP/Type I phosphodiesterase / nucleotide pyrophosphatase;</td></tr>
<tr><td>11</td><td>Tspbctg00000001G00032320.1</td><td>K01519//ITPA`inosine triphosphate pyrophosphatase [EC:3.6.1.-];<br>InP/HAM1;Inosine triphosphate pyrophosphatase/ham1 protein;Ham1 family;ITPase-like;<br>TIGR00042.1//TIGR00042`JCVI: RdgB/HAM1 family non-canonical purine NTP pyrophosphatase;</td></tr>
<tr><td>12</td><td>Tspbctg00000001G00035220.1</td><td>InP/all-alpha NTP pyrophosphatases;Phosphoribosyl-ATP pyrophosphohydrolase;</td></tr>
<tr><td>13</td><td>Tspbctg00000003G00055880.1</td><td>InP/UDP/ADP-Sugar pyrophosphatase;Nucleoside Triphosphate Pyrophosphohydrolase;</td></tr>
<tr><td>14</td><td>Tspbctg00000007G00097730.1</td><td>GO:0010945//CoA pyrophosphatase activity`molecular_function;</td></tr>
<tr><td>15</td><td>Tspbctg00000007G00099540.1</td><td>InP/GTP Pyrophosphatase-related;</td></tr>
<tr><td>16</td><td>Tspbctg00000003G00052790.1</td><td>PF01663.25//Phosphodiest`Type I phosphodiesterase / nucleotide pyrophosphatase;</td></tr>
</table>

<table>
<tr><td>17</td><td>Tspbctg00000005G00075380.1</td><td>GO:0010945//CoA pyrophosphatase activity`molecular_function;</td></tr>
<tr><td>18</td><td>Tspbctg00000005G00081550.1</td><td>PF01663.25//Phosphodiest`Type I phosphodiesterase / nucleotide pyrophosphatase;
InP/Ectonucleotide pyrophosphatase/phosphodiesterase;Type I phosphodiesterase / nucleotide pyrophosphatase;Ectonucleotide pyrophosphatase/phosphodiesterase family member 5;Enpp;</td></tr>
<tr><td colspan="3">Diphosphatase</td></tr>
<tr><td>1</td><td>Tspbctg00000002G00048520.1</td><td>GO:0004170//dUTP diphosphatase activity`molecular_function;</td></tr>
<tr><td>2</td><td>Tspbctg00000002G00045910.1</td><td>TIGR00586.1//mutt`JCVI: 8-oxo-dGTP diphosphatase MutT;</td></tr>
<tr><td>3</td><td>Tspbctg00000003G00054320.1</td><td>K03426//E3.6.1.22, NUDT12, nudC`NAD+ diphosphatase [EC:3.6.1.22];<br>tr|A0A510NVG9|A0A510NVG9_9EURO//NAD(+) diphosphatase {ECO:0000256|ARBA:ARBA00012381} EC=3.6.1.22 {ECO:0000256|ARBA:ARBA00012381} OS=Talaromyces cellulolyticus ORFNames=TCE0_018r05208 {ECO:0000313|EMBL:GAM36256.1} PE=4;<br>GO:0000210//NAD+ diphosphatase activity`molecular_function;</td></tr>
<tr><td>4</td><td>Tspbctg00000003G00058540.1</td><td>tr|A0A0B8N0Z9|A0A0B8N0Z9_9EURO//Inosine triphosphate pyrophosphatase {ECO:0000256|HAMAP-Rule:MF_03148} Short=ITPase {ECO:0000256|HAMAP-Rule:MF_03148} Short=Inosine triphosphatase {ECO:0000256|HAMAP-Rule:MF_03148} EC=3.6.1.9 {ECO:0000256|HAMAP-Rule:MF_03148} Non-canonical purine NTP pyrophosphatase {ECO:0000256|HAMAP-Rule:MF_03148} Non-standard purine NTP pyrophosphatase {ECO:0000256|HAMAP-Rule:MF_03148} Nucleoside-triphosphate diphosphatase {ECO:0000256|HAMAP-Rule:MF_03148} Nucleoside-triphosphate pyrophosphatase {ECO:0000256|HAMAP-Rule:MF_03148} Short=NTPase {ECO:0000256|HAMAP-Rule:MF_03148} OS=Talaromyces cellulolyticus ORFNames=TCE0_038r12254 {ECO:0000313|EMBL:GAM40139.1} PE=3;
GO:0036218//dTTP diphosphatase activity`molecular_function;</td></tr>
<tr><td>5</td><td>Tspbctg00000005G00075900.1</td><td>tr|A0A2H3IKQ6|A0A2H3IKQ6_9EURO//Inorganic diphosphatase {ECO:0000256|ARBA:ARBA00012146} EC=3.6.1.1 {ECO:0000256|ARBA:ARBA00012146} OS=Penicillium sp. 'occitanis' ORFNames=PENO1_079500 {ECO:0000313|EMBL:PCG94245.1} PE=3;
GO:0006796//phosphate-containing compound metabolic process`biological_process;GO:0004427//inorganic diphosphatase activity`molecular_function;</td></tr>
<tr><td>6</td><td>Tspbctg00000001G00030740.1</td><td>GO:0050072//m7G(5')pppN diphosphatase activity`molecular_function;</td></tr>
<tr><td>7</td><td>Tspbctg00000001G00031950.1</td><td>K12584//DCPS, DCS`m7GpppX diphosphatase [EC:3.6.1.59];</td></tr>
<tr><td>8</td><td>Tspbctg00000001G00032310.1</td><td>K12584//DCPS, DCS`m7GpppX diphosphatase [EC:3.6.1.59];</td></tr>
<tr><td>9</td><td>Tspbctg00000001G00032320.1</td><td>GO:0047429//nucleoside-triphosphate diphosphatase activity`molecular_function;</td></tr>
<tr><td>10</td><td>Tspbctg00000001G00035220.1</td><td>GO:0004636//phosphoribosyl-ATP diphosphatase activity`molecular_function;</td></tr>
<tr><td>11</td><td>Tspbctg00000007G00097730.1</td><td>InP/Peroxisomal coenzyme a diphosphatase nudt7;</td></tr>
</table>

12	Tspbctg00000002G00048720.1	InP/8-OXO-Dgtp diphosphates nudt15;Nucleotide triphosphate diphosphatase nudt15;<br>TIGR00586.1//mutt`JCVI: 8-oxo-dGTP diphosphatase MutT;
13	Tspbctg00000005G00075380.1	Nr/KAF3388290.1//Peroxisomal coenzyme A diphosphatase 1, peroxisomal;
14	Tspbctg00000007G00098050.1	tr|A0A2H3I498|A0A2H3I498_9EURO//Guanosine diphosphatase {ECO:0000313|EMBL:PCG88571.1} OS=Penicillium sp. 'occitanis' ORFNames=PENO1_109450 {ECO:0000313|EMBL:PCG88571.1} PE=3;<br>GO:0017110//nucleoside-diphosphatase activity`molecular_function;<br>Ref/XP_002149341.1//nucleoside diphosphatase Gda1;
15	Tspbctg00000000G00005260.1	GO:0017110//nucleoside-diphosphatase activity`molecular_function;<br>InP/GDA1/CD39 family of nucleoside phosphatases signature.;Adenosine/guanosine diphosphatase;GDA1/CD39 (nucleoside phosphatase) family;Nucleoside-diphosphatase MIG-23;Exopolyphosphatase. Domain 2;
**Phosphoesterase**		
1	Tspbctg00000002G00044030.1	PF04185.17//Phosphoesterase`Phosphoesterase family；<br>InP/Phosphoesterase family;
2	Tspbctg00000002G00041030.1	tr|A0A2H3IWV6|A0A2H3IWV6_9EURO//Phosphoesterase {ECO:0000313|EMBL:PCH01904.1} OS=Penicillium sp. 'occitanis' ORFNames=PENO1_040420 {ECO:0000313|EMBL:PCH01904.1} PE=4；<br>PF04185.17//Phosphoesterase`Phosphoesterase family；<br>InP/Phosphoesterase family;
3	Tspbctg00000001G00024240.1	PF00149.31//Metallophos`Calcineurin-like phosphoesterase;<br>InP/Calcineurin-like phosphoesterase;
4	Tspbctg00000005G00074090.1	tr|A0A2H3I4B6|A0A2H3I4B6_9EURO//Phosphoesterase {ECO:0000313|EMBL:PCG93645.1} OS=Penicillium sp. 'occitanis' ORFNames=PENO1_082580 {ECO:0000313|EMBL:PCG93645.1} PE=4；<br>PF04185.17//Phosphoesterase`Phosphoesterase family；<br>InP/Phosphoesterase family;
5	Tspbctg00000002G00046110.1	PF00149.31//Metallophos`Calcineurin-like phosphoesterase;<br>InP/Calcineurin-like phosphoesterase;
6	Tspbctg00000002G00045580.1	PF00149.31//Metallophos`Calcineurin-like phosphoesterase；<br>InP/Calcineurin-like phosphoesterase;
7	Tspbctg00000000G00003290.1	PF00149.31//Metallophos`Calcineurin-like phosphoesterase；<br>InP/Calcineurin-like phosphoesterase;
8	Tspbctg00000007G00095080.1	PF00149.31//Metallophos`Calcineurin-like phosphoesterase；<br>InP/Calcineurin-like phosphoesterase;
9	Tspbctg00000003G00054190.1	InP/DHH phosphoesterases;

<table>
<tr><td>10</td><td>Tspbctg00000000G00012950.1</td><td>PF12850.10//Metallophos_2`Calcineurin-like phosphoesterase superfamily domain；
InP/Calcineurin-like phosphoesterase superfamily domain;yfcE: phosphodiesterase, MJ0936 family;</td></tr>
<tr><td>11</td><td>Tspbctg00000003G00057840.1</td><td>PF04185.17//Phosphoesterase`Phosphoesterase family;
InP/Phosphoesterase family;</td></tr>
<tr><td>12</td><td>Tspbctg00000003G00056860.1</td><td>PF00149.31//Metallophos`Calcineurin-like phosphoesterase;
InP/Calcineurin-like phosphoesterase;</td></tr>
<tr><td>13</td><td>Tspbctg00000001G00025560.1</td><td>PF00149.31//Metallophos`Calcineurin-like phosphoesterase;PF19272.2//ASMase_C`Acid sphingomyelin phosphodiesterase C-terminal region；
InP/Calcineurin-like phosphoesterase;Acid sphingomyelin phosphodiesterase C-terminal region;Sphingomyelin phosphodiesterase;</td></tr>
<tr><td>14</td><td>Tspbctg00000007G00099440.1</td><td>PF04185.17//Phosphoesterase`Phosphoesterase family；
InP/Phosphoesterase family;</td></tr>
<tr><td>15</td><td>Tspbctg00000005G00080830.1</td><td>tr|A0A2H3J0Q3|A0A2H3J0Q3_9EURO//Phosphoesterase {ECO:0000313|EMBL:PCH08592.1} OS=Penicillium sp. 'occitanis' ORFNames=PENO1_006570 {ECO:0000313|EMBL:PCH08592.1} PE=4；
PF04185.17//Phosphoesterase`Phosphoesterase family；
InP/Phosphoesterase family;</td></tr>
<tr><td>16</td><td>Tspbctg00000006G00085240.1</td><td>PF00149.31//Metallophos`Calcineurin-like phosphoesterase；
InP/Calcineurin-like phosphoesterase;</td></tr>
<tr><td>17</td><td>Tspbctg00000001G00035860.1</td><td>PF04185.17//Phosphoesterase`Phosphoesterase family；
InP/Phosphoesterase superfamily protein (AFU_ORTHOLOGUE AFUA_1G17590);Phosphoesterase family;</td></tr>
<tr><td>18</td><td>Tspbctg00000001G00024550.1</td><td>PF00149.31//Metallophos`Calcineurin-like phosphoesterase；
InP/Metallophosphoesterase;MPP_PPP_family;Metallophosphoesterase ynl217w-related;Calcineurin-like phosphoesterase；</td></tr>
<tr><td>19</td><td>Tspbctg00000006G00090540.1</td><td>PF00149.31//Metallophos`Calcineurin-like phosphoesterase；
InP/Calcineurin-like phosphoesterase;</td></tr>
<tr><td>20</td><td>Tspbctg00000002G00040730.1</td><td>PF00149.31//Metallophos`Calcineurin-like phosphoesterase；
InP/Calcineurin-like phosphoesterase;</td></tr>
<tr><td>21</td><td>Tspbctg00000007G00099420.1</td><td>PF00149.31//Metallophos`Calcineurin-like phosphoesterase;
InP/Calcineurin-like phosphoesterase;</td></tr>
<tr><td>22</td><td>Tspbctg00000000G00002870.1</td><td>InP/Calcineurin-like phosphoesterase;</td></tr>
<tr><td>23</td><td>Tspbctg00000002G00045980.1</td><td>PF00149.31//Metallophos`Calcineurin-like phosphoesterase；
InP/Calcineurin-like phosphoesterase;</td></tr>
</table>

24	*Tspbctg00000000G00014100.1*	*KOG3947`Phosphoesterases`General function prediction only；PF00149.31//Metallophos`Calcineurin-like phosphoesterase；InP/Metallophosphoesterase;MPP_239FB;Calcineurin-like phosphoesterase;*
25	*Tspbctg00000005G00080890.1*	*PF00149.31//Metallophos`Calcineurin-like phosphoesterase；InP/Metallophosphoesterase, isoform E;Metallo phosphoesterase related; Calcineurin-like phosphoesterase;*
26	*Tspbctg00000000G00017090.1*	*PF00149.31//Metallophos`Calcineurin-like phosphoesterase; InP/Calcineurin-like phosphoesterase;*
27	*Tspbctg00000001G00037510.1*	*PF00149.31//Metallophos`Calcineurin-like phosphoesterase; InP/Calcineurin-like phosphoesterase; TIGR03729.1//acc_ester`JCVI: putative phosphoesterase (Provisional);*
28	*Tspbctg00000000G00006700.1*	*PF00149.31//Metallophos`Calcineurin-like phosphoesterase; InP/Calcineurin-like phosphoesterase;*
29	*Tspbctg00000004G00072380.1*	*PF00149.31//Metallophos`Calcineurin-like phosphoesterase; InP/Calcineurin-like phosphoesterase;*
30	*Tspbctg00000006G00091290.1*	*KOG3947`Phosphoesterases`General function prediction only; PF00149.31//Metallophos`Calcineurin-like phosphoesterase; InP/Calcineurin-like phosphoesterase;Metallophosphoesterase;*
31	*Tspbctg00000002G00043420.1*	*InP/Metallo phosphoesterase related;*
32	*Tspbctg00000001G00023700.1*	*InP/Calcineurin-like phosphoesterase;*
33	*Tspbctg00000000G00020880.1*	*Nr/KAF3404405.1//Metallophosphoesterase domain-containing protein 1; KOG3947`Phosphoesterases`General function prediction only; PF12850.10//Metallophos_2`Calcineurin-like phosphoesterase superfamily domain;PF00149.31//Metallophos`Calcineurin-like phosphoesterase; InP/Metallophosphoesterase;Calcineurin-like phosphoesterase;*
34	*Tspbctg00000003G00051710.1*	*PF12850.10//Metallophos_2`Calcineurin-like phosphoesterase superfamily domain;PF00149.31//Metallophos`Calcineurin-like phosphoesterase; InP/Metallophosphoesterase ynl217w-related;Metallophosphoesterase; Calcineurin-like phosphoesterase;*
35	*Tspbctg00000004G00069450.1*	*PF00149.31//Metallophos`Calcineurin-like phosphoesterase; InP/Calcineurin-like phosphoesterase;Sphingomyelin phosphodiesterase;*
36	*Tspbctg00000003G00062080.1*	*InP/DNA Ligase D, 3'-Phosphoesterase domain; TIGR02777.1//LigD_PE_dom`JCVI: DNA ligase D, 3'-phosphoesterase domain (Provisional);*

Phosphodiesterase

1	*Tspbctg00000000G00019970.1*	*InP/Type I phosphodiesterase / nucleotide pyrophosphatase;*
2	*Tspbctg00000000G00007080.1*	*K18696//GDE1`glycerophosphodiester phosphodiesterase [EC:3.1.4.46];<br>Nr/KAF3401252.1//Glycerophosphodiester phosphodiesterase GDE1;<br>tr|A0A2H3J4D3|A0A2H3J4D3_9EURO//Glycerophosphocholine phosphodiesterase {ECO:0000256|ARBA:ARBA00024381} EC=3.1.4.2 {ECO:0000256|ARBA:ARBA00024381} OS=Penicillium sp. 'occitanis' ORFNames=PENO1_021970 {ECO:0000313|EMBL:PCH05536.1} PE=4;<br>GO:0047389//glycerophosphocholine phosphodiesterase activity`molecular_function;<br>PF03009.20//GDPD`Glycerophosphoryl diester phosphodiesterase family;<br>InP/Glycerophosphoryl diester phosphodiesterase family;Glycerophosphocholine phosphodiesterase gpcpd1;Phosphatidylinositol (PI) phosphodiesterase;PLC-like phosphodiesterases;Glycerophosphoryl diester phosphodiesterase;*
3	*Tspbctg00000004G00071360.1*	*PF03009.20//GDPD`Glycerophosphoryl diester phosphodiesterase family;<br>InP/PLC-like phosphodiesterases;Glycerophosphoryl diester phosphodiesterase family;Phosphatidylinositol (PI) phosphodiesterase;GP-PDE domain profile.;Glycerophosphoryl diester phosphodiesterase;<br>Ref/XP_046066866.1//putative glycerophosphoryl diester phosphodiesterase;*
4	*Tspbctg00000000G00016040.1*	*tr|A0A2H3J9K6|A0A2H3J9K6_9EURO//Glycerophosphoryl diester phosphodiesterase {ECO:0000313|EMBL:PCH09070.1} OS=Penicillium sp. 'occitanis' ORFNames=PENO1_003460 {ECO:0000313|EMBL:PCH09070.1} PE=4;<br>PF03009.20//GDPD`Glycerophosphoryl diester phosphodiesterase family;<br>InP/GP-PDE domain profile.;GDPD_YPL206cp_fungi;Glycerophosphoryl diester phosphodiesterase;PLC-like phosphodiesterases;Glycerophosphoryl diester phosphodiesterase family;Phosphatidylinositol (PI) phosphodiesterase ;*
5	*Tspbctg00000004G00070370.1*	*K01771//plc`1-phosphatidylinositol phosphodiesterase [EC:4.6.1.13];<br>InP/Phosphatidylinositol (PI) phosphodiesterase;FI02810P;PI-PLCc_BcPLC_like;PLC-like phosphodiesterases;*
6	*Tspbctg00000002G00047150.1*	*GO:0004528//phosphodiesterase I activity`molecular_function;*
7	*Tspbctg00000002G00046510.1*	*K01120//cpdP`3',5'-cyclic-nucleotide phosphodiesterase [EC:3.1.4.17];<br>Nr/KAF3398807.1//3',5'-cyclic-nucleotide phosphodiesterase regA;<br>tr|A0A6V8H9E7|A0A6V8H9E7_9EURO//Phosphodiesterase {ECO:0000256|RuleBase:RU363067} EC=3.1.4.- {ECO:0000256|RuleBase:RU363067} OS=Talaromyces cellulolyticus ORFNames=TCE0_033f08485 {ECO:0000313|EMBL:GAM38050.1} PE=3;<br>GO:0004114//3',5'-cyclic-nucleotide phosphodiesterase activity`molecular_function;<br>KOG3689`Cyclic nucleotide phosphodiesterase`Signal transduction mechanisms;<br>PF00233.22//PDEase_I`3'5'-cyclic nucleotide phosphodiesterase;*

		InP/3'5'-cyclic nucleotide phosphodiesterase domain signature.;3'5'-cyclic nucleotide phosphodiesterase domain profile.;Cyclic nucleotide phosphodiesterase;3'5'-cyclic nucleotide phosphodiesterase;Dual 3',5'-Cyclic-AMP AND -GMP Phosphodiesterase 11;
8	*Tspbctg00000006G00090070.1*	*InP/Phospholipase D phosphodiesterase active site profile.;*
9	*Tspbctg00000000G00000350.1*	*InP/Phospholipase D phosphodiesterase active site profile.;*
10	*Tspbctg00000006G00091810.1*	*K18696//GDE1`glycerophosphodiester phosphodiesterase [EC:3.1.4.46]; Nr/KAH8783734.1//Glycerophosphoryl diester phosphodiesterase family-domain-containing protein; tr|A0A1E1MF38|A0A1E1MF38_RHYSE//Glycerophosphocholine phosphodiesterase {ECO:0000256|ARBA:ARBA00024381} EC=3.1.4.2 {ECO:0000256|ARBA:ARBA00024381} OS=Rhynchosporium secalis (Barley scald fungus) ORFNames=RSE6_08306 {ECO:0000313|EMBL:CZT47712.1} PE=4; GO:0047389//glycerophosphocholine phosphodiesterase activity`molecular_function; InP/Glycerophosphocholine phosphodiesterase GPCPD1;Glycerophosphoryl diester phosphodiesterase;*
11	*Tspbctg00000003G00054720.1*	*PCH07840.1//Type I phosphodiesterase/nucleotide pyrophosphatase/phosphate transferase; tr|A0A2H3IWS6|A0A2H3IWS6_9EURO//Type I phosphodiesterase/nucleotide pyrophosphatase/phosphate transferase {ECO:0000313|EMBL:PCH07840.1} OS=Penicillium sp. 'occitanis' ORFNames=PENO1_010250 {ECO:0000313|EMBL:PCH07840.1} PE=4; PF01663.25//Phosphodiest`Type I phosphodiesterase / nucleotide pyrophosphatase; InP/Type I phosphodiesterase / nucleotide pyrophosphatase;*
12	*Tspbctg00000000G00012950.1*	*InP/yfcE: phosphodiesterase, MJ0936 family; TIGR00040.1//yfcE`JCVI: YfcE family phosphodiesterase;*
13	*Tspbctg00000000G00010020.1*	*tr|A0A2H3IV18|A0A2H3IV18_9EURO//Glycerophosphoryl diester phosphodiesterase {ECO:0000313|EMBL:PCH04850.1} OS=Penicillium sp. 'occitanis' ORFNames=PENO1_025290 {ECO:0000313|EMBL:PCH04850.1} PE=4; PF03009.20//GDPD`Glycerophosphoryl diester phosphodiesterase family; InP/Glycerophosphoryl diester phosphodiesterase;PLC-like phosphodiesterases; Glycerophosphoryl diester phosphodiesterase family;Phosphatidylinositol (PI) phosphodiesterase;*
14	*Tspbctg00000003G00059660.1*	*GO:1903022//positive regulation of phosphodiesterase activity, acting on 3'-phosphoglycolate-terminated DNA strands`biological_process;*
15	*Tspbctg00000003G00056760.1*	*InP/Phospholipase D phosphodiesterase active site profile.;*
16	*Tspbctg00000001G00025560.1*	*K12350//SMPD1, ASM`sphingomyelin phosphodiesterase [EC:3.1.4.12]; tr|A0A2H3I0X9|A0A2H3I0X9_9EURO//Sphingomyelin phosphodiesterase {ECO:0000256|PIRNR:PIRNR000948} OS=Penicillium sp. 'occitanis' ORFNames=PENO1_102300 {ECO:0000313|EMBL:PCG89860.1} PE=3;*

<table>
<tr><td></td><td></td><td>GO:0004767//sphingomyelin phosphodiesterase activity`molecular_function;<br>PF19272.2//ASMase_C`Acid sphingomyelin phosphodiesterase C-terminal region;
InP/Sphingomyelin_Pdiesterase;Acid sphingomyelin phosphodiesterase C-terminal region;Sphingomyelin phosphodiesterase;</td></tr>
<tr><td>17</td><td>Tspbctg00000001G00028560.1</td><td>InP/Phosphatidylinositol (PI) phosphodiesterase;PLC-like phosphodiesterases;</td></tr>
<tr><td>18</td><td>Tspbctg00000001G00027560.1</td><td>InP/Zinc Phosphodiesterase elac protein 2;</td></tr>
<tr><td>19</td><td>Tspbctg00000005G00079540.1</td><td>K18696//GDE1`glycerophosphodiester phosphodiesterase [EC:3.1.4.46];</td></tr>
<tr><td>20</td><td>Tspbctg00000005G00077980.1</td><td>InP/Type I phosphodiesterase / nucleotide pyrophosphatase;</td></tr>
<tr><td>21</td><td>Tspbctg00000001G00030860.1</td><td>K12351//SMPD2`sphingomyelin phosphodiesterase 2 [EC:3.1.4.12];<br>GO:0004767//sphingomyelin phosphodiesterase activity`molecular_function;
InP/Sphingomyelin phosphodiesterase 2;Sphingomyelin phosphodiesterase related;
TIGR03395.1//sphingomy`JCVI: sphingomyelin phosphodiesterase;</td></tr>
<tr><td>22</td><td>Tspbctg00000001G00034340.1</td><td>K01771//plc`1-phosphatidylinositol phosphodiesterase [EC:4.6.1.13];
InP/PLC-like phosphodiesterases;Phosphatidylinositol (PI) phosphodiesterase;</td></tr>
<tr><td>23</td><td>Tspbctg00000005G00084010.1</td><td>InP/Phospholipase D phosphodiesterase active site profile.;</td></tr>
<tr><td>24</td><td>Tspbctg00000001G00034940.1</td><td>GO:0004115//3',5'-cyclic-AMP phosphodiesterase activity`molecular_function;
InP/3',5'-Cyclic-Nucleotide phosphodiesterase 1;3'5'-cyclic nucleotide class II phosphodiesterase signature;cAMP phosphodiesterases class-II;
Ref/XP_002146888.1//cAMP-specific phosphodiesterase, putative;</td></tr>
<tr><td>25</td><td>Tspbctg00000002G00046480.1</td><td>InP/PLC-like phosphodiesterases;Phosphatidylinositol (PI) phosphodiesterase;</td></tr>
<tr><td>26</td><td>Tspbctg00000005G00084950.1</td><td>InP/Cyclic phosphodiesterase;
TIGR02258.1//2_5_ligase`JCVI: RNA 2',3'-cyclic phosphodiesterase;</td></tr>
<tr><td>27</td><td>Tspbctg00000001G00027530.1</td><td>InP/Zinc Phosphodiesterase elac protein 2;</td></tr>
<tr><td>28</td><td>Tspbctg00000001G00033270.1</td><td>Nr/PCG98663.1//RNA ligase/cyclic nucleotide phosphodiesterase;
tr|A0A2H3IIG0|A0A2H3IIG0_9EURO//RNA ligase/cyclic nucleotide phosphodiesterase {ECO:0000313|EMBL:PCG98663.1} OS=Penicillium sp. 'occitanis' ORFNames=PENO1_057030 {ECO:0000313|EMBL:PCG98663.1} PE=4;
InP/Cyclic phosphodiesterase;</td></tr>
<tr><td>29</td><td>Tspbctg00000003G00052790.1</td><td>PF01663.25//Phosphodiest`Type I phosphodiesterase / nucleotide pyrophosphatase;</td></tr>
<tr><td>30</td><td>Tspbctg00000000G00013100.1</td><td>InP/Phosphatidylinositol (PI) phosphodiesterase;PLC-like phosphodiesterases;PI-PLCc_At5g67130_like;</td></tr>
<tr><td>31</td><td>Tspbctg00000003G00049270.1</td><td>InP/Phospholipase D phosphodiesterase active site profile.;</td></tr>
<tr><td>32</td><td>Tspbctg00000005G00077480.1</td><td>Nr/PCG90549.1//Tyrosyl-DNA phosphodiesterase;</td></tr>
</table>

		tr|A0A2H3I091|A0A2H3I091_9EURO//Tyrosyl-DNA phosphodiesterase {ECO:0000313|EMBL:PCG90549.1} OS=Penicillium sp. 'occitanis' ORFNames=PENO1_098640 {ECO:0000313|EMBL:PCG90549.1} PE=3;<br>*KOG2031`Tyrosyl-DNA phosphodiesterase`Replication, recombination and repair;*<br>*PF06087.15//Tyr-DNA_phospho`Tyrosyl-DNA phosphodiesterase;*<br>*InP/Tyrosyl-DNA Phosphodiesterase domain-containing protein;Tyrosyl-DNA Phosphodiesterase 1;Phospholipase D phosphodiesterase active site profile.;Tyrosyl-DNA phosphodiesterase;*
33	*Tspbctg00000002G00040620.1*	*KAF3398799.1//U6 snRNA phosphodiesterase;*<br>*tr|A0A093UXQ6|A0A093UXQ6_TALMA//U6 snRNA phosphodiesterase {ECO:0000256|HAMAP-Rule:MF_03040} EC=3.1.4.- {ECO:0000256|HAMAP-Rule:MF_03040} OS=Talaromyces marneffei PM1 ORFNames=GQ26_0251320 {ECO:0000313|EMBL:KFX45052.1} PE=3;*<br>*InP/Cyclic phosphodiesterase;*
34	*Tspbctg00000005G00081550.1*	*PF01663.25//Phosphodiest`Type I phosphodiesterase / nucleotide pyrophosphatase;*<br>*InP/Ectonucleotide pyrophosphatase/phosphodiesterase;Type I phosphodiesterase / nucleotide pyrophosphatase;Ectonucleotide pyrophosphatase/phosphodiesterase family member 5;Enpp;*
35	*Tspbctg00000004G00069450.1*	*InP/Sphingomyelin phosphodiesterase;*
36	*Tspbctg00000007G00097460.1*	*InP/Phosphatidylinositol (PI) phosphodiesterase;PLC-like phosphodiesterases;*
37	*Tspbctg00000004G00071980.1*	*KOG2814`Transcription coactivator complex, P50 component (LigT RNA ligase/phosphodiesterase family)`Transcription;*<br>*InP/Cyclic phosphodiesterase;*

Note: Gene function annotation database and corresponding abbreviations, KEGG-K; Nr-Nr; Interpro-InP; Refseq-Ref; Pfam-PF; Tigerfam-TIGR; Uniprot-tr; GOGO；KOG-KOG.
