# Supplementary material for: Systematic Investigation of Phosphate Decomposition and Soil Fertility Modulation by the Filamentous Fungus Talaromyces nanjingensis
Source: Microorganisms. 2025 Jul 3;13(7):1574. doi: 10.3390/microorganisms13071574 (PMC12301037; doi:10.3390/microorganisms13071574)
Supplement: Supplementary file 1 [file microorganisms-13-01574-s001.zip › Table S2.pdf]

Tab. S2 Genes associated with environmental adaptability found in *T. nanjingensis* (detailed information version)

Genes and Seq ID		Gene function annotation
**Temperature related**		
Low temperature related		
1	*Tspbctg00000003G00054450.1*	*InP/Hydrophobic protein RCI2 Low temperature and salt responsive protein LTI6 -related;*
2	*Tspbctg00000000G00005140.1*	*InP/Hydrophobic protein RCI2 Low temperature and salt responsive protein LTI6 -related;*
3	*Tspbctg00000004G00070870.1*	*tr|A0A6V8HIJ0|A0A6V8HIJ0_9EURO//Defect at low temperature protein 1 {ECO:0000256|ARBA:ARBA00021353, ECO:0000256|RuleBase:RU367100} OS=Talaromyces cellulolyticus ORFNames=TCE0_042r14928 {ECO:0000313|EMBL:GAM41649.1} PE=3;*<br>*InP/Defect at low temperature protein 1;*
4	*Tspbctg00000000G00020910.1*	*PF06772.14//LtrA`Bacterial low temperature requirement A protein (LtrA);*<br>*InP/Bacterial low temperature requirement A protein (LtrA);BLL5714 PROTEIN;*
5	*Tspbctg00000006G00091970.1*	*InP/Hydrophobic protein RCI2 Low temperature and salt responsive protein LTI6 -related;*
6	*Tspbctg00000003G00056750.1*	*tr|A0A2H3IQH1|A0A2H3IQH1_9EURO//Low temperature viability protein {ECO:0000313|EMBL:PCH03470.1} OS=Penicillium sp. 'occitanis' ORFNames=PENO1_032150 {ECO:0000313|EMBL:PCH03470.1} PE=3;*<br>*PF04180.17//LTV`Low temperature viability protein;*<br>*InP/Low-temperature viability protein ltv1-related;Protein LTV1 homolog;Low temperature viability protein;*
7	*Tspbctg00000002G00044360.1*	*PF06772.14//LtrA`Bacterial low temperature requirement A protein (LtrA);*<br>*InP/Bacterial low temperature requirement A protein (LtrA);BLL5714 Protein;*
8	*Tspbctg00000002G00039940.1*	*Nr/PCG90416.1//Low temperature requirement A;*<br>*tr|A0A2H3I045|A0A2H3I045_9EURO//Low temperature requirement A {ECO:0000313|EMBL:PCG90416.1} OS=Penicillium sp. 'occitanis' ORFNames=PENO1_099330 {ECO:0000313|EMBL:PCG90416.1} PE=4;*<br>*PF06772.14//LtrA`Bacterial low temperature requirement A protein (LtrA);*<br>*InP/Bacterial low temperature requirement A protein (LtrA);*
Cold related		
1	*Tspbctg00000005G00076320.1*	*Nr/KAF3388215.1//Cold-inducible RNA-binding protein;*
High temperature related		
1	*Tspbctg00000003G00055360.1*	*PF12722.10//Hid1`High-temperature-induced dauer-formation protein;*<br>*InP/High-temperature-induced dauer-formation protein;PROTEIN HID1;*
Temperature dependent related		
1	*Tspbctg00000001G00031610.1*	*PF12247.11//MKT1_N`Temperature dependent protein affecting M2 dsRNA replication;PF12246.11//MKT1_C`Temperature dependent protein affecting M2 dsRNA replication;*

		InP/Temperature dependent protein affecting M2 dsRNA replication;H3TH_MKT1;PIN_MKT1;PIN domain-like;Protein MKT1;

Salt related-Salt responsive or salt tolerance

1	*Tspbctg00000003G00054450.1*	*InP/Hydrophobic protein RCI2 Low temperature and salt responsive protein LTI6 -related;*
2	*Tspbctg00000000G00005140.1*	*InP/Hydrophobic protein RCI2 Low temperature and salt responsive protein LTI6 -related;*
3	*Tspbctg00000000G00017760.1*	*PF13945.9//NST1`Salt tolerance down-regulator;*<br>*InP/Stress response protein NST1-related; Stress response protein NST1;*
4	*Tspbctg00000006G00091970.1*	*InP/Hydrophobic protein RCI2 low temperature and salt responsive protein LTI6 -related;*

Resistance related Item type

The number of resistance related items retrieved from the genome of *T. nanjingensis* JP-NJ4 was 541, The following is a brief overview of the types of resistance genes;

1 Antibiotic resistance	*Tetracycline Repressor, domain 2; Tetracycline-efflux transporter, putative; Tetracycline resistance protein, TetA; Tetracycline resistance protein TetB signature; Tetracycline resistance protein signature; Anhydrotetracycline monooxygenase; Brefeldin A-sensitivity protein 4; Brefeldin A sensitivity protein-related; Brefeldin A resistance protein; Bifunctional polymyxin resistance protein arna; beta-Lactam resistance`Human Diseases`Drug resistance: antimicrobial; Efflux pump antibiotic resistance protein; et al;*
2 Antifungal agent resistance	*Leptomycin B resistance protein pmd1; Fluconazole resistance protein 1; et al;*
3 Drug resistance	*Drug resistance protein; multidrug resistance protein (Provisional); Multidrug resistance protein D; et al;*
4 Pesticide resistance	*Cycloheximide resistance protein; et al;*
5 UV radiation resistance	*Ultra violet radiation resistance associated protein; et al;*
6 Disease resistance	*Disease resistance protein; Disease resistance protein signature; et al;*
7 Metal resistance	*Copper resistance protein; Zinc/cadmium resistance protein; Mercury resistance system periplasmic binding protein MerP; et al;*
8 Acid resistance	*Fusaric acid resistance protein-like; et al;*
9 Natural resistance	*Natural resistance-associated macrophage protein;*
10 Other chemical resistance	*Arsenic resistance protein ArsH; Camphor resistance CrcB protein; Nitrosoguanidine resistance protein; Tellurite resistance; Caffeine resistance protein 5; Quinidine resistance protein 1-related; Quinidine resistance protein 2; Quinidine resistance protein 3; Bleomycin resistance protein; Chitin synthesis regulation, resistance to Congo red; et al;*

Siderophore related

Siderophores can be divided into 4 classes depending on its chelation group: carboxylic acid- type, catechol- type, hydroxamate- type and mixed ligand- type Siderophore

1	*Tspbctg00000000G00005630.1*	*Ref/XP_002340383.1//siderophore transcription factor SreA;*

2	*Tspbctg00000006G00087480.1*	*Nr/KAF3397268.1//Siderophore iron transporter mirB;*
3	*Tspbctg00000000G00007690.1*	*Nr/KAF3400842.1//Siderophore iron transporter mirB;*
4	*Tspbctg00000003G00060030.1*	*Nr/KAF3406687.1//Siderophore iron transporter mirB; tr|A0A0B8MYN8|A0A0B8MYN8_9EURO//Siderophore iron transporter {ECO:0000313|EMBL:GAM43274.1} OS=Talaromyces cellulolyticus ORFNames=TCE0_047r17958 {ECO:0000313|EMBL:GAM43274.1} PE=4; Ref/XP_002148447.1//siderophore iron transporter, putative;*
5	*Tspbctg00000006G00086680.1*	*TIGR02718.1//sider_RhtX_FptX`JCVI: RhtX/FptX family siderophore transporter;TIGR00901.1//2A0125`JCVI: AmpG family muropeptide MFS transporter;*
6	*Tspbctg00000007G00099300.1*	*InP/CoA-dependent acyltransferases;Phosphopantetheine attachment site;Acetyl-CoA synthetase-like;Carrier protein (CP) domain profile.;ACP-like;AMP-binding enzyme C-terminal domain;Nonribosomal peptide synthetase;Ferricrocin synthetase (nonribosomal peptide siderophore synthase) (Eurofung);Nonribosomal peptide synthetase, condensation domain;CoA-dependent acyltransferases;AMP-binding enzyme;Putative AMP-binding domain signature.;Condensation domain;*
7	*Tspbctg00000001G00033800.1*	*InP/ACP-like;CT_NRPS-like;CoA-dependent acyltransferases;Phosphopantetheine attachment site.;Hydroxamate-type ferrichrome siderophore peptide synthetase;Putative AMP-binding domain signature.;Nonribosomal peptide synthetase, condensation domain;Acetyl-CoA synthetase-like;Nonribosomal peptide synthetase;Carrier protein (CP) domain profile.;A_NRPS_SidN3_like;AMP-binding enzyme;Condensation domain;AA-adenyl-dom: amino acid adenylation domain; Ref/XP_002146454.1//nonribosomal siderophore peptide synthase, putative;*
8	*Tspbctg00000006G00087520.1*	*InP/Acetyl-CoA synthetase-like;Phosphopantetheine attachment site;Siderophore synthetase (eurofung)-related;FUM14_C_NRPS-like;Luciferase;Domain 3;CoA-dependent acyltransferases;Nonribosomal peptide synthetase;Coil;Nonribosomal peptide synthetase, condensation domain;Condensation domain;AA-adenyl-dom: amino acid adenylation domain;Carrier protein (CP) domain profile.;CT_NRPS-like;AMP-binding enzyme;ACP-like;Putative AMP-binding domain signature.;A_NRPS_SidN3_like;*
9	*Tspbctg00000007G00100710.1*	*InP/Major facilitator superfamily;MFS general substrate transporter;Siderophore iron transporter 1-related;Fungal trichothecene efflux pump (TRI12);MFS general substrate transporter like domains; Ref/XP_002340421.1//siderophore iron transporter, putative;*
10	*Tspbctg00000000G00019610.1*	*Nr/KAF3404989.1//Siderophore iron transporter mirB;*
11	*Tspbctg00000001G00036250.1*	*InP/ACP-like;Carrier protein (CP) domain profile.;Putative AMP-binding domain signature.;A_NRPS_SidN3_like;Coil;Acetyl-CoA synthetase-like;FUM14_C_NRPS-like;Nonribosomal peptide synthetase, condensation domain;CT_NRPS-like;Luciferase;Domain 3;Siderophore synthetase (eurofung)-related;Phosphopantetheine attachment site.;CoA-dependent acyltransferases;Nonribosomal peptide*

<table>
<tr><td></td><td></td><td>synthetase;Condensation domain;AMP-binding enzyme;AA-adenyl-dom: amino acid adenylation domain;</td></tr>
<tr><td>12</td><td>Tspbctg00000001G00032420.1</td><td>tr|A0A6V8H851|A0A6V8H851_9EURO//Siderophore-iron transporter {ECO:0000313|EMBL:GAM35711.1} OS=Talaromyces cellulolyticus ORFNames=TCE0_017r04248 {ECO:0000313|EMBL:GAM35711.1} PE=4;</td></tr>
<tr><td>13</td><td>Tspbctg00000002G00044970.1</td><td>GO:0019290//siderophore biosynthetic process`biological_process;GO:0016746//acyltransferase activity`molecular_function;
Ref/XP_002150260.1//siderophore biosynthesis protein, putative;</td></tr>
<tr><td>14</td><td>Tspbctg00000001G00023950.1</td><td>Nr/KAF3389809.1//Siderophore iron transporter mirB;</td></tr>
<tr><td>15</td><td>Tspbctg00000005G00079520.1</td><td>Ref/XP_002485263.1//siderophore iron transporter, putative;</td></tr>
<tr><td>16</td><td>Tspbctg00000006G00087500.1</td><td>GO:0019290//siderophore biosynthetic process`biological_process;GO:0016746//acyltransferase activity`molecular_function;</td></tr>
<tr><td>17</td><td>Tspbctg00000001G00037440.1</td><td>InP/MFS general substrate transporter;MFS general substrate transporter like domains;Major Facilitator Superfamily;Siderophore iron transporter;MFS general substrate transporter;MFS general substrate transporter like domains;</td></tr>
<tr><td>18</td><td>Tspbctg00000001G00032430.1</td><td>Nr/GAM35711.1//siderophore-iron transporter;
tr|A0A6V8H851|A0A6V8H851_9EURO//Siderophore-iron transporter {ECO:0000313|EMBL:GAM35711.1} OS=Talaromyces cellulolyticus ORFNames=TCE0_017r04248 {ECO:0000313|EMBL:GAM35711.1} PE=4;
InP/MFS general substrate transporter like domains;Siderophore iron transporter 1-related;Major facilitator superfamily;Major facilitator superfamily (MFS) profile.;Major Facilitator Superfamily;MFS general substrate transporter;</td></tr>
<tr><td>19</td><td>Tspbctg00000001G00035980.1</td><td>InP/Siderophore synthetase (eurofung)-related;Carrier protein (CP) domain profile.;Phosphopantetheine attachment site;Coil;A_NRPS_SidN3_like;ACP-like; Acetyl-CoA synthetase-like;Nonribosomal peptide synthetase, condensation domain;CoA-dependent acyltransferases;FUM14_C_NRPS-like;AMP-binding enzyme;Nonribosomal peptide synthetase;Putative AMP-binding domain signature.;Condensation domain;</td></tr>
<tr><td>20</td><td>Tspbctg00000001G00035970.1</td><td>InP/A_NRPS_SidN3_like;Nonribosomal peptide synthetase, condensation domain;Siderophore synthetase (eurofung)-related;Phosphopantetheine attachment site;Putative AMP-binding domain signature.;Acetyl-CoA synthetase-like;ACP-like;Carrier protein (CP) domain profile.;CoA-dependent acyltransferases;FUM14_C_NRPS-like;Acetyl-CoA synthetase-like;AMP-binding enzyme;Nonribosomal peptide synthetase;Condensation domain;</td></tr>
<tr><td colspan="3">Pyoverdine</td></tr>
<tr><td>1</td><td>Tspbctg00000000G00011230.1</td><td>PF05141.15//DIT1_PvcA`Pyoverdine/dityrosine biosynthesis protein;PF02668.19//TauD`Taurine catabolism dioxygenase TauD, TfdA family;
InP/Taurine catabolism dioxygenase TauD, TfdA family;Clavaminate synthase-like;Pyoverdine/dityrosine biosynthesis protein;Biosynthesis protein, putative (afu_orthologue afua_5g02660)-related;</td></tr>
</table>

2	Tspbctg00000000G00019620.1	PF05141.15//DIT1_PvcA`Pyoverdine/dityrosine biosynthesis protein;PF00501.31//AMP-binding`AMP-binding enzyme;<br>InP/AMP-binding enzyme;Putative AMP-binding domain signature.;Acetyl-CoA synthetase-like;ACP-like;Isocyanide synthase A;Pyoverdine/dityrosine biosynthesis protein;Carrier protein (CP) domain profile.;
3	Tspbctg00000000G00004220.1	tr|A0A2H3IN01|A0A2H3IN01_9EURO//Pyoverdine biosynthesis {ECO:0000313|EMBL:PCH06092.1} OS=Penicillium sp. 'occitanis' ORFNames=PENO1_019120 {ECO:0000313|EMBL:PCH06092.1} PE=4;<br>PF05141.15//DIT1_PvcA`Pyoverdine/dityrosine biosynthesis protein;<br>InP/Pyoverdine/dityrosine biosynthesis protein;<br>Ref/XP_002340227.1//pyoverdine/dityrosine biosynthesis protein, putative;
4	Tspbctg00000003G00052030.1	tr|A0A2H3INL2|A0A2H3INL2_9EURO//Pyoverdine biosynthesis {ECO:0000313|EMBL:PCH07323.1} OS=Penicillium sp. 'occitanis' ORFNames=PENO1_012230 {ECO:0000313|EMBL:PCH07323.1} PE=4;<br>PF05141.15//DIT1_PvcA`Pyoverdine/dityrosine biosynthesis protein;PF02668.19//TauD`Taurine catabolism dioxygenase TauD, TfdA family;<br>InP/Taurine catabolism dioxygenase TauD, TfdA family;Pyoverdine/dityrosine biosynthesis protein;Clavaminate synthase-like;Biosynthesis protein, putative (afu_orthologue afua_5g02660)-related;
5	Tspbctg00000000G00004250.1	tr|A0A2H3J092|A0A2H3J092_9EURO//Pyoverdine biosynthesis {ECO:0000313|EMBL:PCH06089.1} OS=Penicillium sp. 'occitanis' ORFNames=PENO1_019090 {ECO:0000313|EMBL:PCH06089.1} PE=4;<br>PF05141.15//DIT1_PvcA`Pyoverdine/dityrosine biosynthesis protein;<br>InP/Pyoverdine/dityrosine biosynthesis protein;Isocyanide synthase A;
Carboxylic acid- type		None
Catechol- type		
1	Tspbctg00000003G00053560.1	GO:0019439//aromatic compound catabolic process`biological_process;GO:0018576//catechol 1,2-dioxygenase activity`molecular_function;GO:0009712//catechol-containing compound metabolic process`biological_process;GO:0008199//ferric iron binding`molecular_function;
2	Tspbctg00000001G00026670.1	GO:0019439//aromatic compound catabolic process`biological_process;GO:0018576//catechol 1,2-dioxygenase activity`molecular_function;GO:0009712//catechol-containing compound metabolic process`biological_process;GO:0008199//ferric iron binding`molecular_function;
3	Tspbctg00000007G00099840.1	GO:0019439//aromatic compound catabolic process`biological_process;GO:0018576//catechol 1,2-dioxygenase activity`molecular_function;GO:0009712//catechol-containing compound metabolic process`biological_process;GO:0008199//ferric iron binding`molecular_function;
Hydroxamate- type		
1	Tspbctg00000001G00033800.1	InP/ACP-like;CT_NRPS-like;CoA-dependent acyltransferases;Phosphopantetheine attachment

site.;Hydroxamate-type ferrichrome siderophore peptide synthetase;Putative AMP-binding domain signature.;Nonribosomal peptide synthetase, condensation domain;Acetyl-CoA synthetase-like;Nonribosomal peptide synthetase;Carrier protein (CP) domain profile.;A_NRPS_SidN3_like;AMP-binding enzyme;Condensation domain;AA-adenyl-dom: amino acid adenylation domain;

Hormone related

Auxin

1 Tspbctg00000001G00027130.1 InP/Auxin efflux transporter family protein (eurofung);Membrane transport protein; TIGR00946.1//2a69`JCVI: auxin efflux carrier;

2 Tspbctg00000003G00053310.1 InP/Membrane transport protein;Auxin efflux transporter family protein (eurofung);Prokaryotic membrane lipoprotein lipid attachment site profile.;

Gibberellin

1 Tspbctg00000006G00085400.1 Ref/XP_002152261.1//gibberellin 20 oxidase, putative;

2 Tspbctg00000006G00089450.1 Ref/XP_002484715.1//gibberellin 3-beta hydroxylase, putative;

Indole-3-acetic acid

1 Tspbctg00000000G00021030.1 GO:0043864//indoleacetamide hydrolase activity`molecular_function;GO:0004040//amidase activity`molecular_function;

2 Tspbctg00000002G00047900.1 GO:0043864//indoleacetamide hydrolase activity`molecular_function;GO:0004040//amidase activity`molecular_function;

3 Tspbctg00000006G00090560.1 GO:0030956//glutamyl-tRNA(Gln) amidotransferase complex`cellular_component;GO:0050567//glutaminyl-tRNA synthase (glutamine-hydrolyzing) activity`molecular_function;GO:0004040//amidase activity`molecular_function;GO:0043864//indoleacetamide hydrolase activity`molecular_function;GO:0005739//mitochondrion`cellular_component; GO:0070681//glutaminyl-tRNAGln biosynthesis via transamidation`biological_process;GO:0032543//mitochondrial translation`biological_process;GO:0005524//ATP binding`molecular_function;

4 Tspbctg00000001G00034920.1 GO:0043864//indoleacetamide hydrolase activity`molecular_function;GO:0004040//amidase activity`molecular_function;

1-aminocyclopropane-1-carboxylate deaminase (ACC deaminase)

1 Tspbctg00000005G00079470.1 K01505//E3.5.99.7`1-aminocyclopropane-1-carboxylate deaminase [EC:3.5.99.7]; Nr/KAF3390871.1//putative 1-aminocyclopropane-1-carboxylate deaminase; tr|A0A2H3IL07|A0A2H3IL07_9EURO//1-aminocyclopropane-1-carboxylate deaminase {ECO:0000313|EMBL:PCG96085.1} OS=Penicillium sp. 'occitanis' ORFNames=PENO1_070140 {ECO:0000313|EMBL:PCG96085.1} PE=3; GO:0008660//1-aminocyclopropane-1-carboxylate deaminase activity`molecular_function;GO:0030170//pyridoxal

		phosphate binding`molecular_function;GO:0009310//amine catabolic process`biological_process; InP/ACCD;ACCD_DCysDesulf;Pyridoxal-phosphate dependent enzyme;Tryptophan synthase beta subunit-like PLP-dependent enzymes;1-Aminocyclopropane-1-carboxylate deaminase-related;ACC_deam: 1-aminocyclopropane-1-carboxylate deaminase; Ref/XP_002487215.1//1-aminocyclopropane-1-carboxylate deaminase, putative; TIGR01274.1//ACC_deam`JCVI: 1-aminocyclopropane-1-carboxylate deaminase;TIGR01275.1//ACC_deam_rel`JCVI: pyridoxal phosphate-dependent enzymes, D-cysteine desulfhydrase family (Provisional);
2	*Tspbctg00000000G00019590.1*	*Nr/KAF3404991.1//1-aminocyclopropane-1-carboxylate synthase-like protein 1; InP/1-aminocyclopropane-1-carboxylate synthase signature;Aminotransferase class I and II;Aminotransferases class-I pyridoxal-phosphate attachment site.;Bifunctional aspartate aminotransferase and glutamate/aspartate-prephenate aminotransferase-related;Putative (afu_orthologue afua_4g00630)-related;PLP-dependent transferases;AAT_like;Aspartate Aminotransferase, domain 1;*
3	*Tspbctg00000005G00084710.1*	*Nr/GAM37438.1//1-aminocyclopropane-1-carboxylate oxidase; tr|A0A6V8HGL5|A0A6V8HGL5_9EURO//1-aminocyclopropane-1-carboxylate oxidase {ECO:0000313|EMBL:GAM37438.1} OS=Talaromyces cellulolyticus ORFNames=TCE0_024r07359 {ECO:0000313|EMBL:GAM37438.1} PE=3;*
4	*Tspbctg00000005G00081680.1*	*InP/Bifunctional aspartate aminotransferase and glutamate/aspartate-prephenate aminotransferase-related;Putative (afu_orthologue afua_4g00630)-related;Aminotransferase class I and II;PLP-dependent transferases;Aminotransferases class-I pyridoxal-phosphate attachment site.;AAT_like;1-aminocyclopropane-1-carboxylate synthase signature;Putative (afu_orthologue afua_4g00630)-related;*
5	*Tspbctg00000000G00017450.1*	*tr|A0A6V8HE67|A0A6V8HE67_9EURO//1-aminocyclopropane-1-carboxylate oxidase {ECO:0000313|EMBL:GAM39573.1} OS=Talaromyces cellulolyticus ORFNames=TCE0_034f11233 {ECO:0000313|EMBL:GAM39573.1} PE=3;*
6	*Tspbctg00000000G00008640.1*	*tr|A0A6V8H003|A0A6V8H003_9EURO//1-aminocyclopropane-1-carboxylate oxidase {ECO:0000313|EMBL:GAM34126.1} OS=Talaromyces cellulolyticus ORFNames=TCE0_015r01499 {ECO:0000313|EMBL:GAM34126.1} PE=4;*
Cytokinin (zeatin)/ Ethylene/Abscisic acid		*None*
Strigolactone/Brassinosteroid		*None*
**Other growth-regulating substances related**		
Jasmonic acid/jasmonate		*None*
Salicylicacid/salicylate		The number of salicylicacid related items retrieved from the genome of *T. nanjingensis* JP-NJ4 was 71

Polyamine	The number of polyamine related items retrieved from the genome of *T. nanjingensis* JP-NJ4 was 144

Note: Gene function annotation database and corresponding abbreviations, KEGG-K; Nr-Nr; Interpro-InP; Refseq-Ref; Pfam-PF; Tigerfam-TIGR; Uniprot-tr; GO-GO；KOG-KOG. None is marked in the Table if no relevant information is retrieved from the genome annotation.
