# Supplementary figures and images for: Systematic Investigation of Phosphate Decomposition and Soil Fertility Modulation by the Filamentous Fungus Talaromyces nanjingensis
Source: Microorganisms. 2025 Jul 3;13(7):1574. doi: 10.3390/microorganisms13071574 (PMC12301037; doi:10.3390/microorganisms13071574)

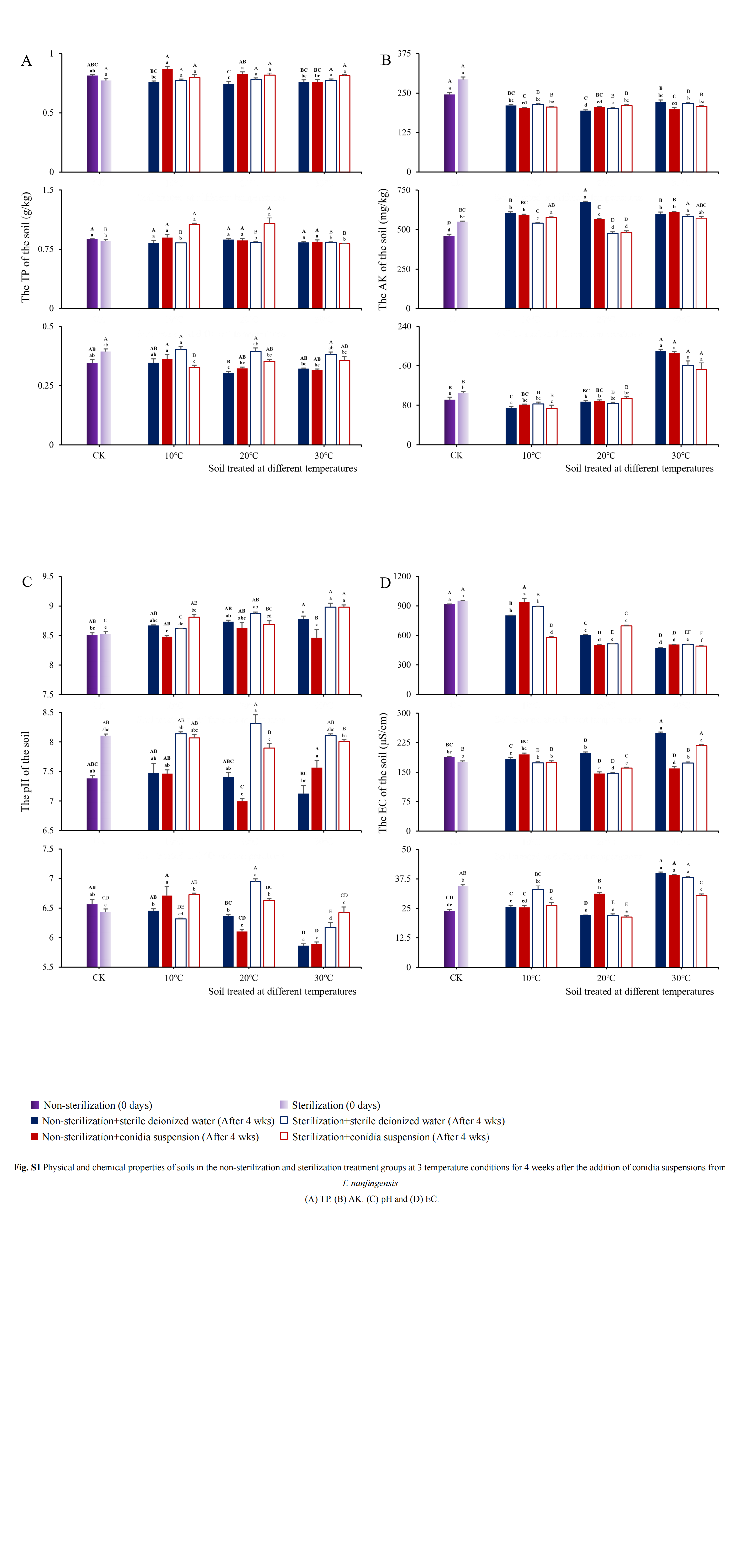

Supplement: Supplementary file 1 [file microorganisms-13-01574-s001.zip › Figure S1.tif]
